# Riboflavin along with antibiotics balances reactive oxygen species and inflammatory cytokines and controls *Staphylococcus aureus* infection by boosting murine macrophage function and regulates inflammation

**DOI:** 10.1186/s12950-016-0145-0

**Published:** 2016-11-28

**Authors:** Somrita Dey, Biswadev Bishayi

**Affiliations:** Department of Physiology, Immunology Laboratory, University of Calcutta, University Colleges of Science and Technology, 92 A.P.C. Road, Calcutta, 700009 West Bengal India

**Keywords:** Murine peritoneal macrophage, Pro-inflammatory cytokines, Reactive oxygen, Species, Riboflavin, *S. aureus*

## Abstract

**Background:**

Macrophages serve as intracellular reservoirs of *S. aureus*. Recent in vitro studies have confirmed high level resistance by *S. aureus* to macrophage mediated killing and the intracellular persistence of Staphylococci may play an important role in the pathogenesis. Since this localization protects them from both cell-mediated and humoral immune responses, therefore, a successful anti-staphylococcal therapy should include the elimination of intracellular bacteria, further protecting the host cells from staphylococci-induced cell death. So, only antibiotic therapy may not be helpful, successful therapy needs combination of drugs not only for elimination of pathogen but also for rescuing the host cell for *S. aureus* induced cell death.

**Methods:**

In keeping with this idea an in vitro study has been done to examine the effect of Riboflavin along with antibiotics on phagocytosis, hydorgen peroxide, superoxide production, antioxidant enzyme levels, and cytokine levels in mouse macrophages for amelioration of the *Staphylococcus aureus* burden. The immune boosting effects of Riboflavin have been validated through perturbations of redox homeostasis and pro-inflammatory cytokines measurements.

**Results:**

It was observed that the supplementation of Vitamin B-2 (Riboflavin) not only enhances macrophage function as previously reported but also decreases pro-inflammatory responses in *Staphylococcus aureus* infected macrophages. The observed influence of Riboflavin on enhanced antimicrobial effects such as enhanced phagocytosis of macrophages exposed to *S. aureus*, hydrogen peroxide or superoxide production when combined with either ciprofloxacin (CIP) or Azithromycin (AZM) and decrease in pro-inflammatory responses of IFN-γ, IL-6, IL-1β. Riboflavin treatment also decreased NO and TNF-α level possibly by inhibiting the NF-κβ pathway. The increased antioxidant enzymes like glutathione reductase, SOD and GSH level helped in maintaining a stable redox state in the cell.

**Conclusion:**

Riboflavin plus antibiotic pretreatment not only enhances macrophage functions but also decreases proinflammatory responses in *Staphylococcus aureus* infected macrophages indicating better bacterial clearance and regulated inflammation which may be considered as a novel and important therapeutic intervention.

## Background

Phagocytic effecter function is the basic to host defense against intracellular pathogens such as *Staphylococcus aureus* (*S. aureus*), the gram positive coccal bacteria, the etiological agent of local infections (e.g., wound infection, furuncle and cellulitis) to systemic dissemination (bacteremia) and finally to metastatic infections (e.g. endocarditis, osteomyelitis and septic arthritis) [1]. Microbes upon entrance to host body are destined to engulfment by professional phagocytes such as neutrophils, macrophages and dendritic cells. Among few organisms, *S.aureus* possess diverse mechanism to avoid destruction in phagolysosomes [2]. Recent in-vitro studies revealed greater resistance of *S.aureus* to killing by macrophages [3]. Upon internalization by macrophages, *S. aureus* is widely assumed to be confined within phagosome following its maturation and fusion with endosomes and lysosomes, creates an inhospitable environment for invading microorganisms, boosting acidification, and augmentation of ROS, and other charged anti-microbial peptides [4]. *S. aureus* has evolved a diversified array of antioxidant tools both enzymatic and non-enzymatic to resist immune mediated oxidative attack [5].

With the emergence of MRSA (methicillin resistant *S. aureus*) strains with reduced susceptibility to vancomycin, and as there are numerous discrepancies for antibiotics action, starting from the degree of plasma membrane permeability, segregation to diverse compartments, to inactivation by intracellular environment [6]. Thus there is the need of combinational therapy with efficacious antibiotics having intracellular bactericidal activity, being harsher to pathogen and least severe to host cells. Thus, different antibiotics or drugs amalgamation can implement successive anti staphylococcal mission and further not only reduce infection but can also aid in protection of host cell from *Staphylococcus aureus* infection induced cell death.

Riboflavin (vitamin B-2) is an essential micronutrient found in a large variety of foods. Vitamin B-2 is necessary for maintaining proper functioning of the nervous, endocrine, cardiovascular and immune systems. Riboflavin is known to elevate immune functions by activation of macrophages, conferring bactericidal action from its spectrum of actions. Riboflavin participates in numerous diverse internal redox reactions as a part of metabolism. An inadequate intake of this vitamin would contribute to difficulties in intermediary metabolism [7]. Riboflavin kinase (RFK) in cell converts Riboflavin into flavin mononucleotide (FMN) and flavin adenine dinucleotide (FAD), which are essential cofactors of dehydrogenases, reductases, and oxidases including the phagocytic NADPH oxidase 2 (Nox2) and participate in wide range of redox reactions [8, 9]. Riboflavin as a proteasome inhibitor quenches inflammation by reduction of proinflammatory cytokines [10]. Moreover, in-vivo treatment of mice with Riboflavin reduces the mortality of mice with septic shock [11] and enhances the resistance to bacterial infections [12].

Imbalance between Reactive oxygen species (ROS) and antioxidant enzymes confers to cytotoxicity, and thus balance between these two ensures prevention from chronic diseases [13]. ROS produced by NADPH oxidase (NOX) envisage its role as defense and signaling molecules related to innate immunity and various other cellular responses [14]. In the early innate immune response H_2_O_2_ kills bacteria through classic ROS respiratory burst. Superoxide anion does not diffuse across the membranes efficiently and is rapidly dismuted to H_2_O_2_ by superoxide dismutase (SOD). However, H_2_O_2_ can diffuse more freely and causes direct oxidative damage to many pathogens. NOX-2 derived ROS is the main, but not the only source of oxidative attack on invading organism [15] and are critical components for host defense against bacterial infection [16]. As the balance in the levels and rates of production of ROS and NO dictates oxidative versus nitrosative stress, these differences may be crucial in understanding how immune responses are regulated in host cells upon treatment with Riboflavin and antibiotics during *S. aureus* infection. Cells contain several anti-oxidant systems to protect themselves from the injury induced by increased intracellular ROS. However the effect of antibiotics along with Riboflavin was not investigated in infection induced oxidative stress and inflammation in macrophages [17].

Azithromycin (AZM), the macrolide antibiotic has the potential to accumulate inside the cell and therefore have an intracellular responsiveness [18]. Intracellular azithromycin enhances phagocytic killing of staphylococci. Azithromycin has the ability to concentrate within neutrophils, and dynamically act against intracellular organisms [19]. Ciprofloxacin (CIP) which belongs to group of fluoroquinolones is potent synthetic agents active against a variety of bacterial species in vitro. Fluroquinolone antibiotics accumulation in mammalian PMNs can be largely found intracellularly than extracellularly. Further, it has also been seen that enhanced trapping of antibiotic is observed in infected phagocytic cell and thus more clearance of bacteria.

Other than antimicrobial activity, antibiotics are also potent immuno-modulators [20]. They are capable of reducing the production of pro-inflammatory cytokines like IL-1β, IL-6, IL-8, TNF-α, IFN-γ and also regulate anti-inflammatory IL-10 in the cytokine milieu during acute phase inflammatory processes [21, 22]. Furthermore, antibiotics diminish the release of various oxidizing species like superoxide anion and nitric oxide that take part in the innate immunity of the host phagocytic cells, their prominent modulatory effect on several nuclear transcription factors such as NF-κB and activator protein-1 (AP-1) in the cell cytosol has been documented. [23].

But as already mentioned, although advancement antibiotic dosing regimen, the increasing prevalence of infections caused by multidrug-resistant bacteria is a global health problem that has been exacerbated by the dearth of novel classes of antibiotics. Herein, we tried combination therapies for the treatment of multidrug-resistant bacterial infections using an in vitro infection model. These efforts include antibiotic–antibiotic combinations, such as AZM and CIP along with the use of certain other agents like Riboflavin, which could delineate *S. aureus* burden by activation of macrophage weaponry against the pathogen by enhanced production of ROS and cytokines, the therapeutic approach of which will provide adequate coverage for potential pathogens causing infections.

The present study is a contribution to the current knowledge about oxidative stress caused by *S. aureus* infection in macrophages, suggesting that an investigation of ROS, NO and antioxidant enzymes should be performed in order to detect the contribution of infection induced oxidative stress and inflammation and its amelioration by treatment of host cells with Riboflavin and antibiotics including CIP and AZM. In response to the regulated production of NO and pro inflammatory cytokines, there was a rise in the activity of SOD and enzymes of the Glutathione system and decrease in the expression of COX-2, the enzyme which drives acute inflammation from its onset to its resolution was investigated upon combinational treatment. Production of pro-inflammatory cytokines was regulated by combined treatment with Riboflavin and antibiotics. Thus, this combinational therapy could bestow protection to the host cell and ultimately restrict *S. aureus* infection induced cell death.

## Methods

### Maintenance of animals

Animals were maintained and experiments were conducted as accredited by the Institutional Animal Ethics Committee (IAEC), Department of Physiology, University of Calcutta, under the guidance of CPCSEA (PROPOSAL NO: IAEC-III/PROPOSAL/BB/02/2013 dated 24.04.2013) Ministry of Environment and Forest, Government of India. Wild type male Swiss albino mice 6–8 weeks old, weighing 20 ± 4 gm was used for the study and was maintained in quarantine for 1 week. They were randomized into plastic cages with filter bonnets and saw dust bedding. Mice were fed a normal rodent diet and tap water ad libitum. Animal holding rooms were maintained at 21 to 24 °C and 40–60% humidity with a 12-h light dark cycle. Before surgery mice were anaesthetized with inhaling anesthetics (ether). Euthanasia was performed by general anesthesia followed by vital tissue removal using 2–3% ether for induction and 1% for maintenance.

### Preparation of bacteria

The *Staphylococcus aureus* strain AG-789 was obtained from Apollo Gleneagles Hospital, Calcutta, West Bengal, India. The bacteria were cultured in Muller Hinton broth (MHB) purchased from Hi-media and were grown overnight. Freshly prepared culture was centrifuged so as to collect bacterial cells from mid logarithmic growth phase. The cell pellets were then re- suspended with sterile 0.9% PBS and was diluted to reach an optical density (O.D.) of 0.2 at 660 nm with the help of a spectrophotometer. An O.D. of 0.2 at 660 nm corresponds to 5 × 10^7^ cells/ml *S.aure*us and the colony forming unit (CFU) count of the desired inoculum was confirmed by serial dilution and culture on blood agar plates [24].

### Isolation of murine peritoneal macrophages

Intra peritoneal injection of 4% sterile thioglycolate broth, 2 ml of volume was administered in mice. After 4 to 5 days, the resulting peritoneal exudate was harvested by lavage of the peritoneal cavities of mice with endotoxin-free Hanks’ solution. Peritoneal macrophages were suspended in 0.83% ammonium chloride solution containing 10% (v/v) Tris buffer (pH 7.65) to lyse erythrocytes. The cells were re-suspended in RPMI 1640 medium supplemented with 10% FBS, 100 IU/ml penicillin, and 100 μg/ml streptomycin, and then were incubated for 1 h at 37 °C on plastic surface. Non-adherent cells were removed by aspiration and washing with RPMI 1640 medium before the addition of *S. aureus*. The adherent cells of which more than 95% were found macrophages as determined by light microscope and were used for in vitro experiments [25].

### Assay for quantification of riboflavin inside the macrophage

Murine Peritoneal macrophages (5×10^6^ cells/ml) were mixed with *S. aureus* (5×10^6^ CFU/ml) in a 1:1 cell: bacterium ratio [26] in RPMI-FBS (5%) and incubated at 37 °C cell culture incubator in presence of antioxidant Riboflavin (100 μg/mL) and antibiotics AZM or CIP for 60 and 90 mins. Cells were pelleted down by centrifugation and washed with PBS. Cells were lysed using 0.1% BSA and absorbance was measured at 440 nm using a UV-Visible spectrophotometer and was compared with a standard curve of Riboflavin, then Riboflavin quantity inside the cell was analyzed.

### Assay for intracellular killing

The presence of a large number of *S. aureus* bacteria in some cells that had undergone in vitro culture observed in the bacterial plate count suggests that the bacteria grow intracellularly. In addition, it has been reported that *S. aureus* multiplies in the presence of intact macrophages in cell cultures and would not grow in macrophage-conditioned media. Therefore, the number of *S. aureus* bacteria grown in a petri dish after time-dependent phagocytosis indicates the number of *S. aureus* bacteria that have survived inside the macrophages after ingestion. For this, murine peritoneal macrophages (5×10^6^ cells/ml) were mixed with *S. aureus* (5×10^6^ CFU/ml) in a 1:1 cell: bacterium ratio [26] in RPMI-FBS (5%) and incubated at 37 °C cell culture incubator for different times in presence or absence of antioxidant Riboflavin (100 μg/mL) and antibiotics Azithromycin (AZM) and Ciprofloxacin (CIP). Phagocytosis was stopped by adding cold (4 °C) RPMI-1640 and extracellular *S. aureus* were removed by washing the suspension in RPMI, note that we were unable to use antibiotics to kill bacteria present outside of macrophages because engulfed bacteria died quickly during the period necessary for the action of antibiotics. Centrifugation was performed and cell culture supernatants were collected and stored for further assay. The pellets were disrupted in sterile water containing 0.01% bovine serum albumin (BSA) by vigorously vortexing to release intracellular bacteria. The lysates containing bacteria were plated at serial dilutions on mannitol agar plates. The plates were incubated at 37 °C for a day or two and the number of colonies was determined [20].

### Study of immunofluorescence for engulfment of bacteria by peritoneal macrophages and their co-localization by confocal microscopy

The engulfment of *S. aureus* by murine peritoneal macrophages was analyzed by confocal microscopy. The merged image, where macrophages were in blue and internalized bacteria was in green indicated that the internalized bacteria were brightly fluorescent after engulfment within peritoneal macrophages. Macrophages were cultured, and the cells were re-suspended in RPMI 1640 medium supplemented with 10% FBS, 100 IU/ml penicillin, and 100 μg/ml streptomycin. At 18 h prior to infection, 5×10^5^ cells were seeded in six-well plates (total volume 3 ml) (Invitrogen). Macrophages cell nuclei were stained using Nuclear yellow (Hoechst S769121) (ab13903) at 250 μg/ml dilution for 2 h. For the FITC staining of *S. aureus,* bacteria were grown overnight at 37 °C in MHB broth. Then the bacteria were diluted with fresh broth and were cultured until mid-logarithmic phase of growth (OD_600_ = 0.3). Bacteria were harvested, washed with PBSE (PBS + 5 mM EDTA) and adjusted to 1x10^9^ CFU/ml. Bacterial pellets of 1×10^9^ CFU were re-suspended in carbonate buffer (pH = 9.0) containing 100 μg/ml Fluorescein Isothiocyanate (FITC) isomer I (Abcam, catalog No: ab 145325) for 1 h at room temperature. Staphylococci were extensively washed with PBSE.

Prior to infection, cells were washed with growth medium without antibiotics and kept for 1 h at 37 °C. A total of 10^6^ to 10^8^ FITC-stained staphylococci were added per well. Extracellular staphylococci were killed by incubation of cells with lysostaphin (Sigma) for 7 min at 37 °C. After the invasion procedure, cells were rinsed with PBS and fixed for 15 min with 4% para-formaldehyde. Specimens were mounted on microscopy slides in 10% glycerol and visualized in 60× oil immersion lens. Excitation and emission filter for FITC is 495/519 nm and for Hoechst is 355/495 nm respectively. Images were acquired by using an Olympus Laser Confocal Scanning Microscope (Spectral type inverted Microscope IX81) and then analyzed by using Olympus FV1000 viewer software. Co-localization was accessed by Pearson correlation coefficient [26].

### Assay for quantification of hydrogen peroxide (H_2_O_2_) production

Activation of leukocytes by inflammatory stimuli results in the local release of ROS and induces hydrogen-peroxide (H_2_O_2_) production. Studies in animals support a critical role for phagocyte oxidative burst in controlling *S. aureus* infection. Therefore, the quantification of H_2_O_2_ in this experimental setup was quite relevant.

After time-dependent phagocytosis, cell lysates were prepared from the pellet. H_2_O_2_ assay from the lysate was performed according to the method as described earlier with slight modification [25]. Briefly 70 μl of lysate, 20 μl Horse Raddish peroxidase (HRP) (500 μg/ml), 70 μl of Phenol red (500 μg/ml) and 40 μl medium were added in each well of the micro-titer plate and was allowed for incubation for 2 h at 37 °C. The reaction was stopped by adding 25 μl of 2 (N) NaOH and the absorbance reading was taken at 620 nm. Control set received 40 μl of HBSS in place of supernatant/lysate. A standard curve for H_2_O_2_ was plotted and the amount of H_2_O_2_ released in the lysate was evaluated and expressed in μM/10^6^cells.

### Assay for quantification of superoxide anion (O_2_^−^) production

Because intracellular killing is dependent on oxidants, it was expected that the amount of superoxide anion released by the host macrophages also contributes to oxidant-dependent killing of *S. aureus*. Since superoxide anion production has been implicated in many physiological and pathological processes, including host innate immune and inflammatory responses to pathogens, we were also interested in determining the amount of superoxide anion released in this experimental setup. Superoxide anion release assay measures the change in colour of Cytochrome C (cytC), when reduced by O_2_- released from the stimulated macrophages. Lysates were incubated in presence of Cytochrome C (100 μl at 2 mg/ml). The production of superoxide anion was monitored spectrophotometrically at 550 nm with reference to the blank. The amount of superoxide anion production was calculated by the following formula [25]: Nano moles of superoxide anion = (mean absorbance at 550 nm × 15.87).

### Determination of intracellular ROS by flow cytometry

The choice of a sensitive method for detection of intracellular ROS is very important for detecting the disturbed redox balance inside the cells. Flow cytometric data acquisition and analysis were performed on BD FACS Verse. The flow cytometer was equipped with an iron laser with excitation at 488 nm and 15 mW output power. A single cell suspension was prepared having a density of 10^5^–10^6^ cells/ml and a minimum of 10,000 cells were measured. Non fluorescent 2,7-dichlorofluorescin diacetate (DCFH-DA) was commonly employed which upon esterase activity within the cells converts to DCFH which may react with several ROS including hydrogen peroxide, hydroxyl radicals and peroxynitrite and get converted to a fluorescent product (DCF). The amount of DCF was proportional to the amount of ROS present inside the cell. Briefly, 20 mM DCFHDA stock solution made in DMSO was diluted in the cultured medium from different groups of macrophages, to yield 20 mM working solution. Then, the cells were incubated for 60 min in the dark at 37 °C. Finally, cells were suspended in PBS, and kept on ice for immediate detection of intracellular ROS generation at 530 nm [27].

### Assay for quantification of nitric oxide (NO) production

The amount of NO released by the macrophages was determined by the Griess assay. 50 μl of lysate was incubated separately in 40 μM Tris (pH = 7.9) containing 40 μM of the reduced form of β-Nicotinamide Adenine Dinucleotide Phosphate, 40 μM Flavin Adenine Dinucleotide and 0.05 U/ml nitrate reductase at 37 °C for 15 min. Reduced samples were incubated with an equal volume of Griess reagent consisting of sulphanilamide; 0.25% (w/v) and N-1-naphthylethylenediamine; 0.025% (w/v), the mixture was incubated for 10 mins then the absorbance at 550 nm was measured. The total nitrate/nitrite concentration was determined by comparison to a reduced NaNO_3_ standard curve [25].

### Assay of catalase enzyme activity

Typically, *S. aureus*-stimulated leukocytes produce proinflammatory cytokines, which trigger ROS production in the tissues through NADPH oxidase activation. Thus, the level of antioxidant enzymes or their activity displays the intracellular complex mechanisms of the host’s defense. Therefore, to determine the activity of these antioxidant enzymes in neutralizing the ROS molecules produced, we estimated the antioxidant enzyme activity in the supernatant or cell-free lysate of macrophages after infection in the presence or absence of Riboflavin or antibiotics. Catalase activity in the cell free lysate was determined spectrophotometrically by measuring the decrease in H_2_O_2_ concentration at 440 nm. At time zero, 100 μl of the supernatant or cell free lysate was added separately to 2.89 ml of potassium phosphate buffer (pH 7.4) taken in a quartz cuvette. To it 0.1 ml of 300 mM H_2_O_2_ was added and absorbance was taken at 240 nm for 5 min at 1 min intervals. Catalase activity was expressed in terms of mmole/min mg protein [20].

### Assay of superoxide dismutase (SOD) enzyme activity

In addition to blocking phagolysosomal acidification, subsequent treatment of macrophages with antibiotics might regulate intracellular ROS and can also protect bacteria from oxidative stress by modulating bacterial superoxide dismutase (SOD) expression or catalase activity. So estimation of SOD enzyme activity in this experimental setup was seemed helpful to get an idea of ROS level.100 μl of the lysate was mixed separately with 1.5 ml of a Tris-EDTA-HCl buffer (pH 8.5), then 100 μl of 7.2 mmol/L pyrogallol was added and the reaction mixture was incubated at 25 °C for 10 min. The reaction was terminated by the addition of 50 μl of 1M HCI and measured at 420 nm. One unit was determined as the amount of enzyme that inhibited the oxidation of pyrogallol by 50%. The activity was expressed as U/mg protein [20].

### GSH assay

SOD, catalase enzymes, and glutathione (GSH) are important scavengers of the superoxide anion and hydrogen peroxide. These enzymes prevent the generation of the hydroxyl radical and protect the cellular constituents from oxidative damage. SOD rapidly dismutates superoxide anion to H_2_O_2_, which is further degraded by catalase into water and oxygen. It was demonstrated that a decrease in the concentration of cellular GSH indicates oxidative damage due to stress or inflammation. So estimation of GSH level was also rational in this setup. Reduced glutathione content (as acid soluble sulfhydryl) was estimated by its reaction with DTNB (Ellman’s reagent) following the method of Sedlac and Lindsey with some modifications. 0.3 ml of sample was mixed with 0.3 ml 10% TCA followed by vortexing. Then the mixture was centrifuged at 5000 rpm for 10 min at 40 °C. To 250 μl of the resultant supernatant, 500 μl 0.8M Tris–HCl was added followed by addition of 25 μl of 5, 5′ - dithiobis-2-nitrobenzoic acid (DTNB). The absorbance was measured at 412 nm using a UV–VIS spectrophotometer to determine GSH content. The values were expressed as nmoles of GSH per mg protein [20].

### Lipid peroxidation (LPO) assay

Elevation of LPO level was considered as an indicator of oxidative stress. Lipid peroxidation of the lysates was determined as Thio- barbituric acid reactive substances (TBARS). In brief, the lysate was mixed with trichloro acetic acid-thiobarbituric acid-hydrochloric acid (TBA-TCA-HCl) reagent and mixed thoroughly and heated for 20 min at 80 °C. The tubes containing the samples were then cooled to room temperature. The absorbance of the pink chromogen present in the clear supernatant after centrifugation at 1200×g for 10 min at room temperature was measured at 532 nm using a UV-VIS spectrophotometer. Tetraethoxy-propane was used as standard. The values were expressed as nmoles of TBARS per mg protein [13].

### Assay of glutathione reductase enzyme activity

The glutathione reductase activity was determined following the oxidation of NADPH to NADP+ during the reduction of oxidized glutathione. The reaction was initiated by the addition of cell lysate in a quartz cuvette containing 1.5 ml reaction mixture of 0.3 mM of NADPH and 3 mM oxidized glutathione in 0.2 M k_2_HPO_4_ buffer (pH = 7.5). The decrease in absorbance at 340 nm was followed for 3 mins on a spectrophotometer and the glutathione reductase activity was calculated using 6.22, the millimolar extinction coefficient for NADPH at 340 nm. The activity glutathione reductase enzyme was expressed as nmol NADPH/min/mg of protein [28].

### Tumor necrosis factor alpha (TNF-α), interferon gamma (IFN-γ), interleukin-6 (IL-6), Interleukin 1 beta (IL1-β), interleukin-10 (IL-10) and MCP-1 ELISA assays

As high ratio of IL-10 to TNF-α was associated with fatal outcome in patients with infection, we focused our study on the production of pro-inflammatory cytokines and an anti-inflammatory cytokine. Cytokines such as IFN- γ, IL-1β and IL-6 reportedly play a protective role in host resistance to facultative intracellularly growing bacteria. IL-10 is a potent anti-inflammatory cytokine and inhibits the synthesis of pro-inflammatory cytokines from TH1 cells, which have a suppressive effect on TNF-α, IFN-γ and IL-12 production. Cytokine concentrations from cell culture supernatants were determined by sandwiched ELISA. For this in vitro study, supernatants from different groups were normalized to the protein content by Lowry method before the assay and the levels of four major pro- inflammatory cytokines TNF-α, IFN-γ, IL-6, IL-1β along with an anti-inflammatory cytokine IL-10 and chemokine MCP-1 were determined as per manufacturer’s guidelines of Ray biotech, Inc, USA in a Bio-Rad ELISA Reader at 450 nm. The minimum detectable value of TNF-α was <60 pg/mL, IFN-γ was <5 pg/mL, IL-6 < 2 pg/mL, IL-10 < 45 pg/mL, IL-1β < 5 pg/ml and MCP-1 was <3 pg/ml as given in the manual. The reproducibility of cytokine kits are: intra-assay: CV <10%, inter assay: CV <12%.

### Western blot analysis for cyclooxygenase-2 (COX-2) expression

Given its constitutively expressed nature and predominant role in prostaglandin synthesis during bacterial infection, potential strategies for drug resistant bacteria based on COX pathways or inhibiting COX-2 is a potential target. These data might support that combinatorial antibiotic and Riboflavin treatment mediated COX-2 inhibition or strategies that disrupt prostaglandin signaling pathways as useful adjunctive therapies in treating persistent and multi-drug resistant infections. Murine macrophages after treatment with different combinations as described earlier were lysed with RIPA-NP40 buffer containing 0.5 mM PMSF, 1 mM sodium orthovanadate, and 1 mg/mL protein inhibitor cocktail (1 mg/mL leupeptin, 1 mg/mL aprotonin, 10 mg/mL soybean trypsin inhibitor, 1 mg/mL pepstatin) and normalized to the protein content by Lowry method. Samples containing equal amounts of protein in equal volumes of sample buffer were separated in a denaturing 10% polyacrylamide gel and transferred to a 0.1 μm nitrocellulose membrane. Nonspecific binding sites were blocked with Tris-buffered saline (TBS; 40 mM Tris, pH 7.6, 300 mM NaCl) containing 5% nonfat dry milk for 1 h at room temperature. Membrane was then incubated with primary antibody to COX-2 (1:500 dilution) (catalogue number: orb106537; Biorbyt Limited, UK). Blots were washed three times in TBST (TBS with 0.1% Tween 20), incubated for 2 h with appropriate horseradish peroxidase-conjugated secondary antibody, developed with the Super Signal chemiluminescent substrate (Thermo Scientific, USA) and exposed to X-Omat BT films (Kodak). Beta-tubulin was used as loading control to ensure equal loading of samples throughout the gel [25].

### Statistical analysis

Isolated peritoneal macrophages from mice were pooled together to obtain the requisite amount of macrophages (5 × 10^6^ cells/ml), and the different parameters were measured. This was repeated for three times for each parameter (e.g., H_2_O_2_ production), then the mean value of these triplicate experiments was taken for calculation. Data was expressed as mean ± SD. Means were compared between groups by using analysis of variance (ANOVA). *P* < 0.05 was considered significant.

## Results

### Riboflavin intake by *S. aureus* infected peritoneal macrophages inside the cell

Prior treatment of mouse peritoneal macrophages with riboflavin before *S. aureus* infection showed significantly more Riboflavin content inside the cell in comparison to only Riboflavin pretreated macrophages at 60 and 90 mins post infection (*p* < 0.05). The Riboflavin content inside the macrophages were further increased when in addition to Riboflavin cells were treated with AZM or CIP prior to *S. aureus* infection (Table 1).

**Table 1:** Riboflavin concentration inside the macrophages (μg/ml)

Samples	Concentrations of riboflavin uptake (μg/ml)	
	60 min	90 min
CM + RIBO	37.31724 ± .551	38.21379 ± .482
RIBO + SAM	46.48966 ± 1.724^a^	46.35172 ± 1.655^a^
RIBO + AZM + SAM	46.48966 ± 1.724^a^	54.97241 ± .1379^b^
RIBO + CIP + SAM	52.76552 ± 0.689^b^	57.31724 ± .413^b^

Riboflavin concentrations inside the macrophages were estimated from the cell lysate by spectrophotometric analysis and were expressed in μg/ml. Results were shown as mean ± SD of three independent experiments

*CM + RIBO* Riboflavin pre-treated Control macrophage, *RIBO + SAM*, Riboflavin pretreated + *S. aureus* infected macrophages, *RIBO + AZM + SAM* Riboflavin and Azithromycin pretreated macrophages infected with *S. aureus*, *RIBO + CIP + SAM* Riboflavin and Ciprofloxacin pretreated macrophages infected with *S. aureus*

^a^significant difference with respect to CM + RIBO

^b^significant difference with respect to Riboflavin (RIBO+SAM)

### Effect of Riboflavin and antibiotic treatment either alone or in combination on phagocytosis of *S. aureus* by peritoneal macrophage

**Fig. 1:**
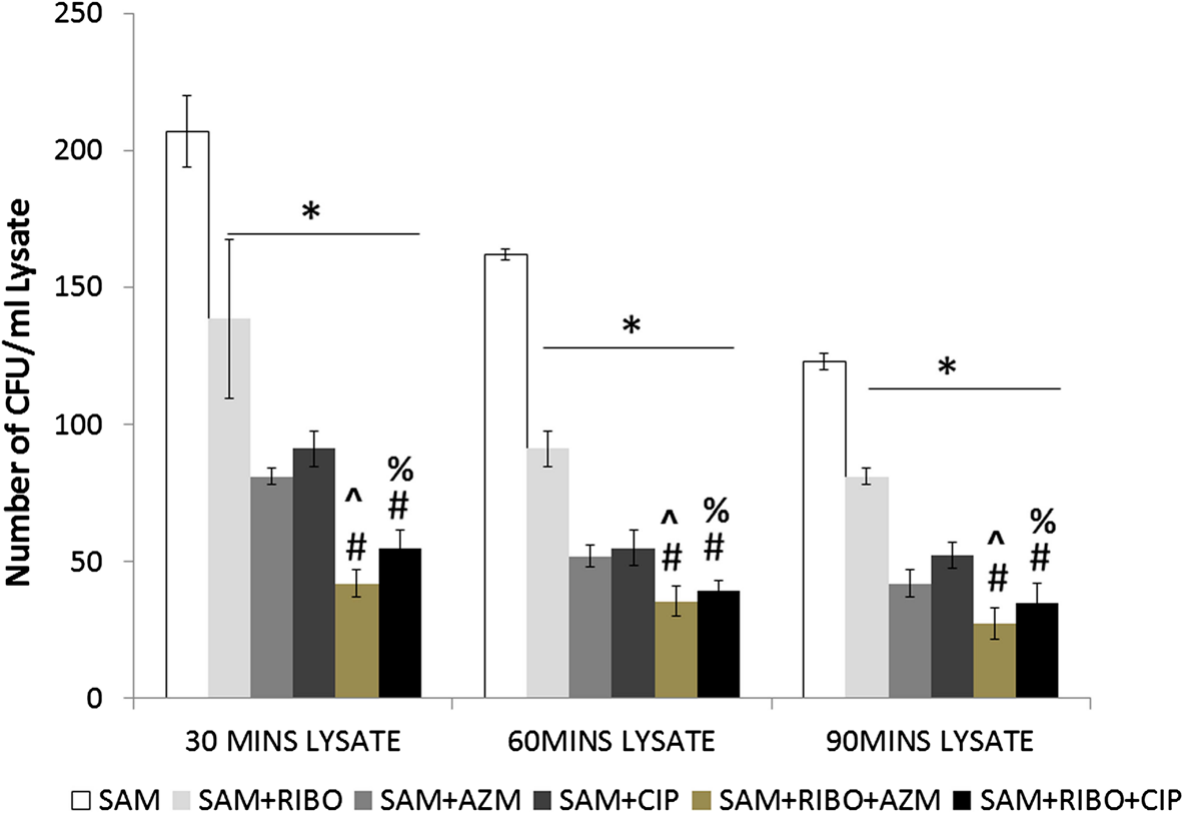
Effect of Riboflavin and antibiotic treatment on the intracellular killing of *S. aureus infected* murine peritoneal macrophages. Intracellular killing assay was determined from the lysate after time dependent phagocytosis in presence or absence of Riboflavin or antibiotics and was expressed in terms of number of CFU/ml lysate. Results were presented as mean ± SD of three independent experiments. &, significant difference with respect to CM; *, significant difference with respect to SAM; #, significant difference with respect to Riboflavin(RIBO) plus *S. aureus* infected macrophages at *P* < 0.05 level of significance.^, significant difference with respect to ciprofloxacin (CIP) or % Significant difference with respect to Azithromycin (AZM) plus *S. aureus* infected macrophages

The intracellularly viable bacterial count (CFU) was significantly (*p* < 0.05) lessened at 30, 60 and 90 min post infection when the murine peritoneal macrophages were pre incubated with Riboflavin and compared to the *S. aureus* infected macrophages at 30, 60 and 90 mins (Fig. 1). The phagocytic activity of peritoneal macrophages were further increased upon treatment with Riboflavin along with antibiotics AZM or CIP for 1 h prior to live *S. aureus* infection, whereas maximum phagocytic activity or least CFU was found at 90 mins when *S. aureus* infected macrophages were co-treated with Ciprofloxacin in combination with Riboflavin and compared to only *S. aureus* infected macrophages at *p* < 0.05 level of significance (Fig. 1).

### Study of immune-fluorescence for engulfment of bacteria by peritoneal macrophages and their co-localization by confocal microscopy

**Fig. 2:**
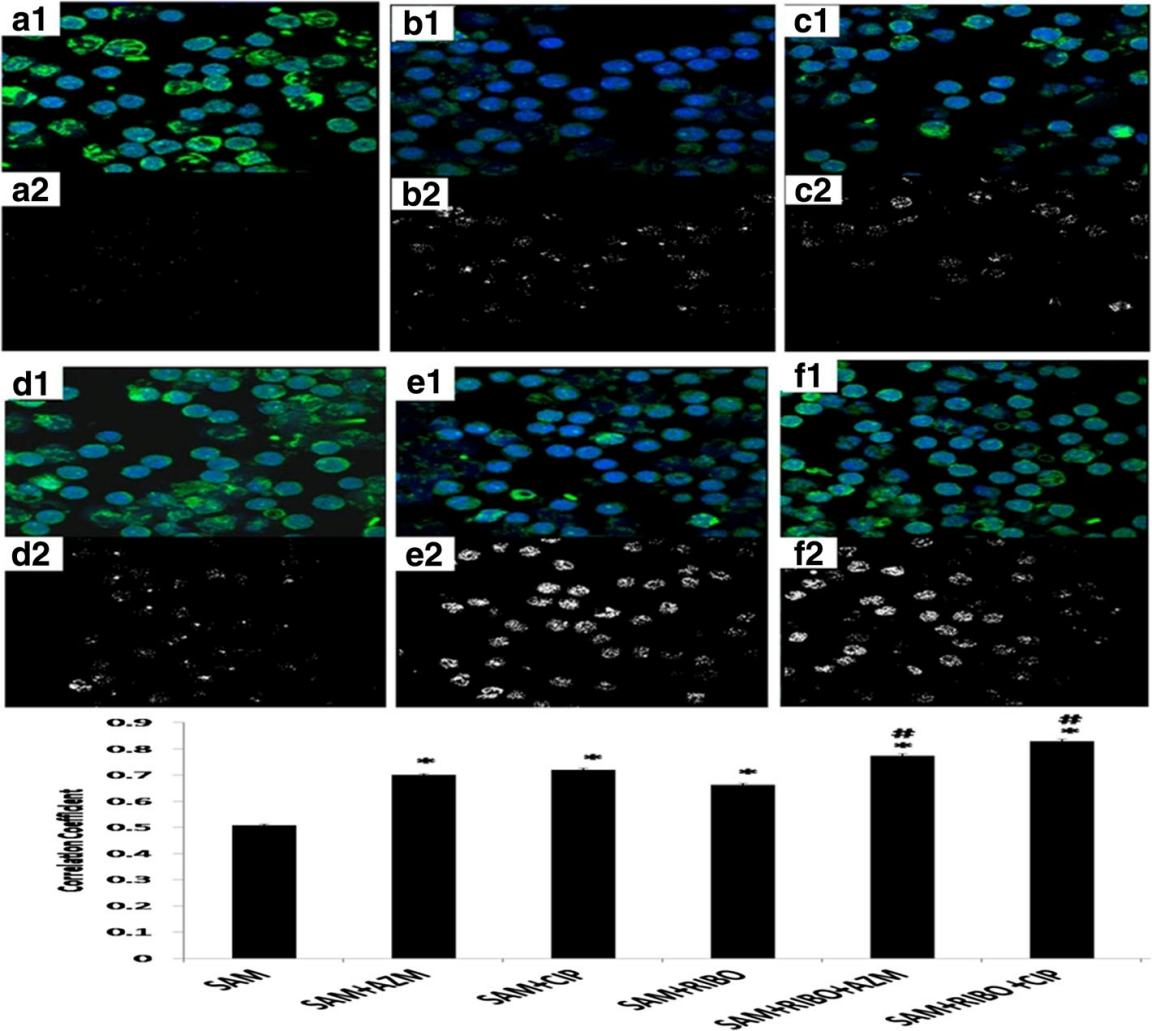
Study of immunofluorescence for engulfment of bacteria by peritoneal macrophages and their co-localization by confocal microscopy. Phagocytosis analysis was done by confocal microscopy. In Fig. 2 (a1, b1, c1, d1, e1, f1), the merged image of phagocytosis was depicted, where green (FITC) stained bacteria were phagocytosed by Hoechst stained (*Blue*) macrophage. Fig. 2(A2, B2, C2, D2, E2, F2) represent signals from co-localized probes. Fig. 2G Pearson’s correlation coefficients (PCCs) of images of internalized FITC stained *S.aureus* in Hoechst stained Riboflavin either alone or AZM or CIP pre-treated Macrophage cells. Fig. 2 (A1, A2) represents SAM, Fig. 2 (B1, B2): AZM + SAM, Fig. 2 (C1, C2): CIP + SAM, Fig. 2 (D1, D2) :RIBO + SAM, Fig. 2 (E1, E2): RIBO + AZM + SAM, Fig. 2 (F1, F2): RIBO + CIP + SAM. *, represents significant difference with respect to SAM,#, represent significant difference with respect to RIBO + SAM at *p* < 0.05 level of significance

Bacterial infection experiments or internalization of *S. aureus* in the murine peritoneal macrophages in presence of Riboflavin and antibiotics were further analyzed by confocal microscopic imaging. Results showed engulfment of *S. aureus* by murine peritoneal macrophages at 90 mins of incubation (Fig. 2). From the Pearson’s Co localization coefficient it was found that significantly increased engulfment as evident from more co-localized signals when macrophages were pre-treated with Riboflavin before *S. aureus* infection and compared to only *S. aureus* infected macrophages (Fig. 2a, d). Co-localized signals were found to be significantly (*p* < 0.05) increased when *S. aureus* infected macrophages were treated with AZM or CIP and compared to only *S. aureus* infected macrophage (Fig. 2a, b, c). Furthermore, significantly (*p* < 0.05) increased co-localized signals was also observed when macrophages were further treated with AZM or CIP along with Riboflavin and compared to SAM and RIBO + SAM groups (Fig. 2d, e, f), which corresponds to more engulfment and lower colony forming unit in phagocytosis assay.

### Effect of Riboflavin and antibiotic treatment on *S. aureus* infection-induced hydrogen peroxide production by murine peritoneal macrophages

**Fig. 3:**
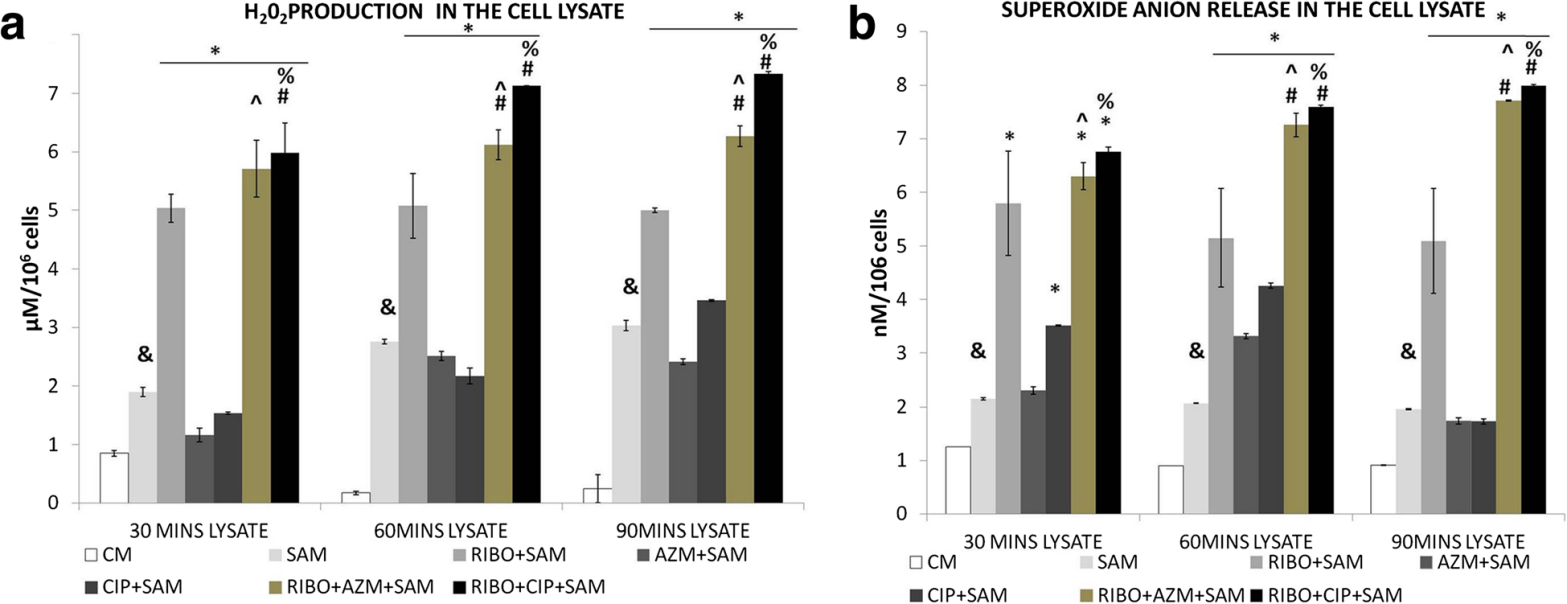
Effect of Riboflavin and antibiotic treatment on *S. aureus* infection induced alteration in the H_2_O_2_ release and superoxide anion release in the lysate of murine peritoneal macrophages. The lysate recovered after time dependent phagocytosis in presence or absence of Riboflavin or antibiotics were used to determine the H_2_O_2_ (**a**) and superoxide anion release (**b**) and was expressed in terms of μM/10^6^cell and nM/10^6^cells. Results were presented as mean ± SD of three independent experiments. CM, Control macrophage; SAM, *S. aureus* infected macrophages; RIBO + SAM, Riboflavin pretreated + *S. aureus* infected macrophages; AZM + SAM, Azithromycin treated *S.aureus* infected macrophage; CIP + SAM, Ciprofloxacin treated *S.aureus* infected macrophage; RIBO + AZM + SAM, Riboflavin and Azithromycin pretreated macrophages exposed to *S. aureus*. RIBO + CIP + SAM, Riboflavin and Ciprofloxacin pretreated macrophages exposed to *S. aureus*; &, significant difference with respect to CM *, significant difference with respect to SAM; #, significant difference with respect to Riboflavin(R) plus *S. aureus* infected macrophages at *P* < 0.05 level of significance.^, significant difference with respect to ciprofloxacin (CIP) or % Significant difference with respect to Azithromycin (AZM) plus *S. aureus* infected macrophages

We tested whether the used antibiotics as well as Riboflavin has any role in hydrogen peroxide production by peritoneal macrophages during *S. aureus* infection. Our results showed (Fig. 3a) that the increase in the amount of H_2_O_2_ released in the lysate by live *S. aureus* infected peritoneal macrophages was significant at 60 and, 90 mins post infection when compared to control macrophage. When control macrophages were treated with Riboflavin, a significant increase in H_2_O_2_ production at 60 and 90 mins post infection was observed (Table 2). When *S. aureus* infected macrophages were incubated in presence of Riboflavin along with Azithromycin or Ciprofloxacin, a significant (*p* < 0.05) H_2_O_2_ release was noticed when compared to SAM as well as RIBO + SAM. However, Ciprofloxacin along with Riboflavin treated macrophages showed maximal H_2_O_2_ production.

**Table 2:** Determination of H_2_O_2_, Superoxide anion and nitric oxide (NO) released from different control sets

Groups	H_2_O_2_ (μM/10^6^cell)			Superoxide anion (nM/10^6^cells)			Nitric oxide (mM/10^6^cells)		
	30MINS	60MINS	90MINS	30MINS	60MINS	90MINS	30MINS	60MINS	90MINS
Control Mφ	0.85 ± 0.05	0.17 ± 0.03	0.24 ± 0.02	1.25 ± 0.004	0.90 ± 0.002	0.91 ± 0.002	0.69 ± 0.002	0.71 ± 0.001	0.53 ± 0.018
Control Mφ + RIBO	1.16 ± 0.1^a^	2.41 ± 0.05^a^	2.51 ± 0.08	1.7 ± 0.06^a^	1.96 ± 0.01^a^	2.31 ± 0.0^a^	0.530 ± 0.018	0.45 ± 0.00^a^	0.40 ± 0.001
Control Mφ + AZM	0.77 ± 0.01	0.53 ± 0.01^a^	0.17 ± 0.03	1.16 ± 0.12	1.58 ± 0.02^a^	2.07 ± 0.002^a^	0.32 ± 0.017^a^	0.53 ± 0.01	0.77 ± 0.01^a^
Control Mφ + CIP	1.16 ± 0.1^a^	1.54 ± 0.02^a^	1.90 ± 0.08^a^	1.25 ± 0.004	0.96 ± 0.01	2.15 ± 0.02^a^	0.72 ± 0.002^a^	0.85 ± .0.05	0.913 ± 0.01^a^

H_2_O_2_, superoxide anion and NO were determined from the control macrophages either alone or in combination with RIBO, AZM or CIP. Results were shown as mean ± SD of three independent experiments

*CM* Control macrophage, *CM + RIBO* Riboflavin pre-treated Control macrophage, *AZM + CM* Azithromycin pretreated control macrophage, *CIP + CM* Ciprofloxacin pretreated control macrophage

^a^significant difference with respect to CM

### Effect of Riboflavin and antibiotic treatment on *S. aureus* infection-induced superoxide anion (O_2_^−^) production by murine peritoneal macrophages

Our results showed (Fig. 3b) significant increase in the O_2_^−^ released in the lysate by live *S. aureus* infected peritoneal macrophages at 60 and 90 mins post infection when compared to control macrophages. When the control macrophages were treated with Riboflavin a significant increase in O_2_^−^ production was found (Table 2). When *S. aureus* infected macrophages were incubated in presence of Riboflavin along with Azithromycin and Ciprofloxacin, a significant (*p* < 0.05) increment in superoxide anion release was noticed when compared to SAM at 60 mins and RIBO + SAM groups at 60 and 90mins respectively. Among these Ciprofloxacin along with Riboflavin treated macrophages showed maximal O_2_^−^ production.

### Effect of Riboflavin and antibiotic treatment on *S. aureus* infection-induced cellular ROS production by murine peritoneal macrophages

**Fig. 4:**
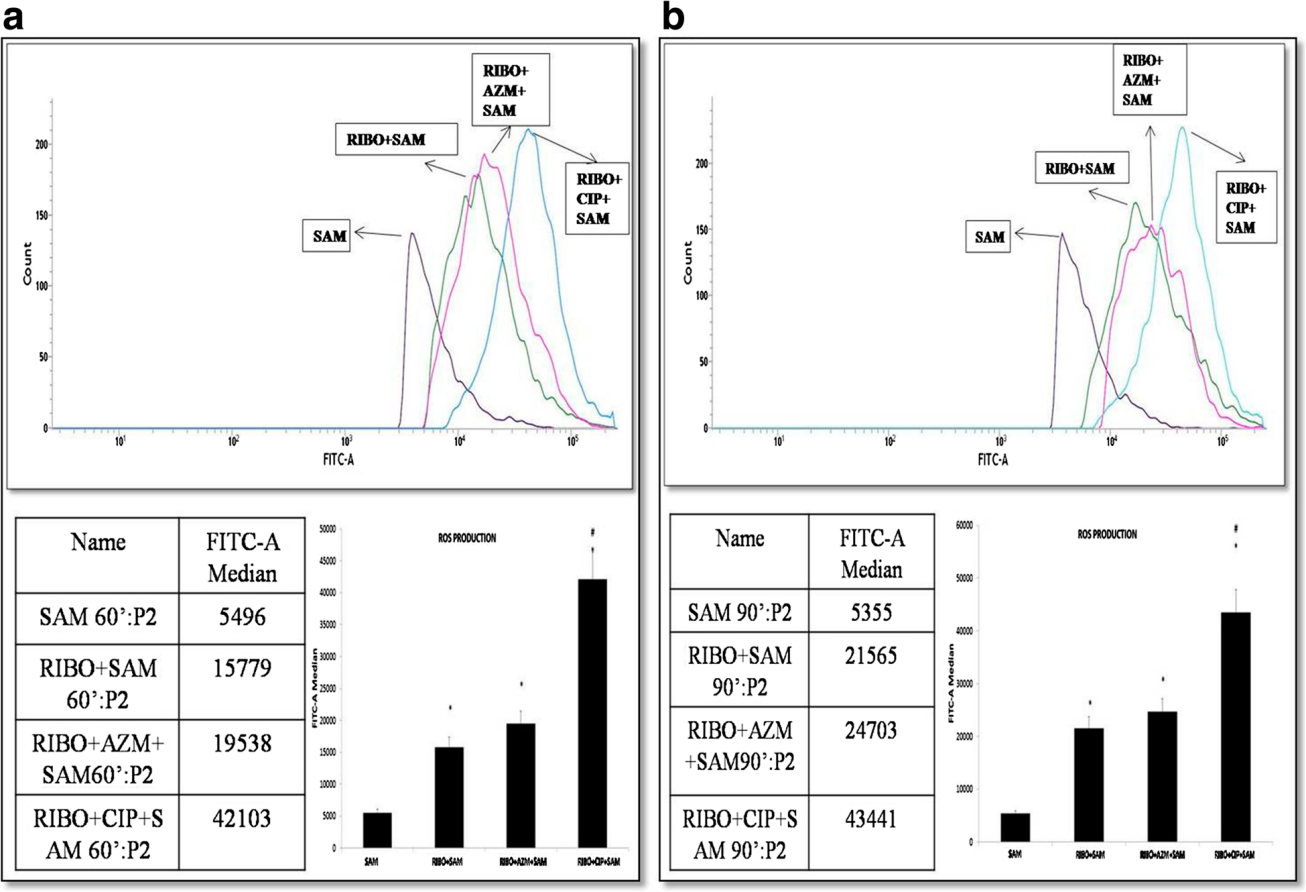
Effect of Riboflavin and antibiotics AZM and CIP on intracellular ROS production by murine peritoneal macrophages **a** represents production of ROS after 60minutes of incubation **b** represents production of ROS after 90 minutes of incubation. Peritoneal macrophages were treated with Riboflavin either alone or in combination with AZM or CIP and exposed to *S. aureus* and were incubated with 20 μM DCHF-DA for 60 min. Fluorescence from cells was measured by Flow Cytometry. Results were presented as mean ± SD of three independent experiments. *, significant difference with respect to SAM; #, significant difference with respect to Riboflavin (RIBO) plus *S. aureus* infected macrophages at *P* < 0.05 level of significance

From the hydrogen peroxide and super oxide free radical production, it was observed that treatment of macrophages with Riboflavin either alone or in combination with AZM or CIP after *S. aureus* infection led to significant increase (*p* < 0.05) in hydrogen peroxide and super oxide anion production, we further determined the intracellular ROS production by flow cytometry at 60 and 90 min after infection. Following the incubation with DCF-DA, it was observed that the macrophages which were treated with Riboflavin and exposed to *S. aureus* (Fig. 4a, b) showed a marked increase (*p* < 0.05) in ROS production than that of non Riboflavin treated groups. In addition to it, a significant increase (*p* < 0.05) in ROS production was also observed at 60 mins (Fig. 4a) and 90 mins (Fig. 4b) when *S. aureus* infected peritoneal macrophages were treated with CIP or AZM along with Riboflavin and compared to only Riboflavin treated macrophages. Among these the FITC-A median value was found to be highest when macrophages were treated with RIBO and CIP before *S.aureus* infection which depicts highest ROS production.

### Effect of Riboflavin and antibiotic treatment on *S. aureus* infection-induced nitric oxide (NO) production by murine peritoneal macrophages

**Fig. 5:**
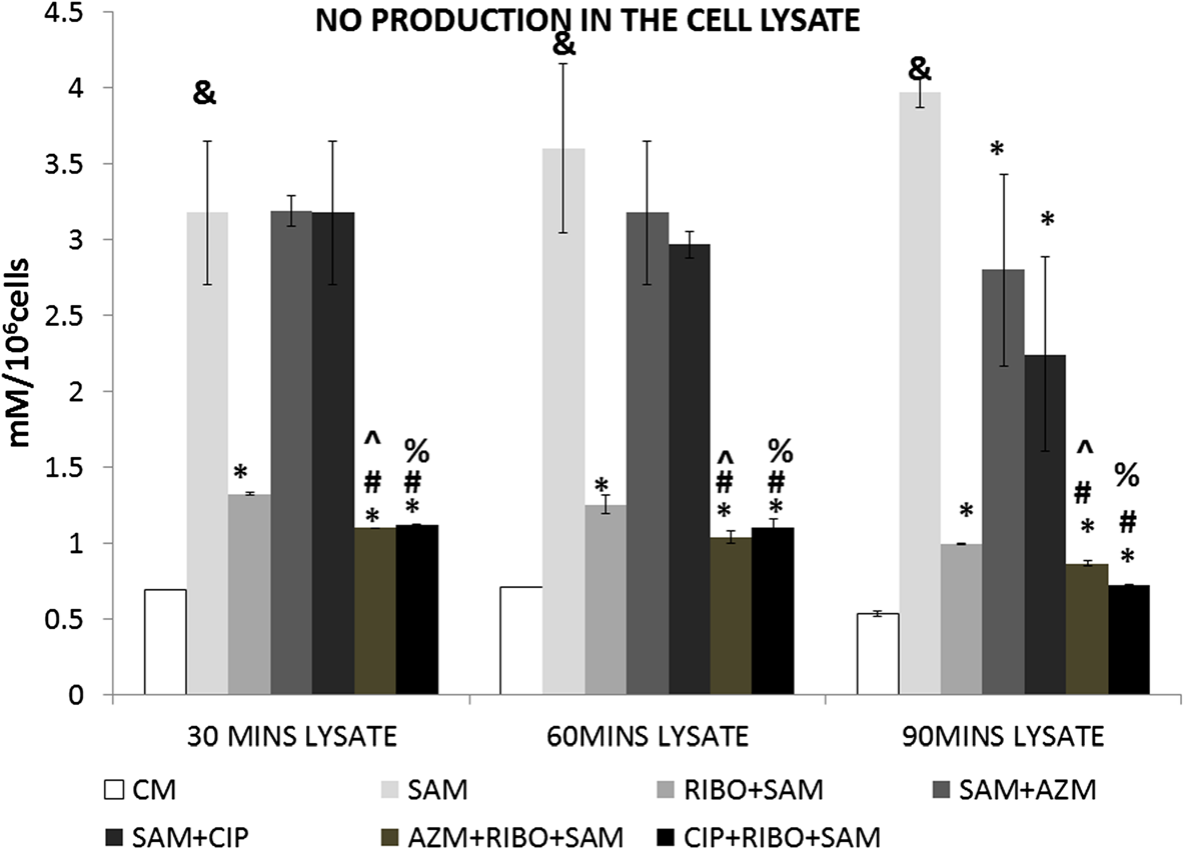
Effect of Riboflavin and antibiotic treatment on *S. aureus* infection induced nitric oxide release in the supernatant and lysate of murine peritoneal macrophages. The lysate recovered after time dependent phagocytosis in presence or absence of Riboflavin or antibiotics were used to determine the nitric oxide (NO) release by the Griess assay and were expressed in terms of mM/10^6^cells. Results were presented as mean ± SD of three independent experiments. &, significant difference with respect to CM *, significant difference with respect to SAM; #, significant difference with respect to Riboflavin (RIBO) plus *S. aureus* infected macrophages at *P* < 0.05 level of significance.^, significant difference with respect to ciprofloxacin (CIP) or % Significant difference with respect to Azithromycin (AZM) plus *S. aureus* infected macrophages

The amount of NO released in the lysate respectively at 30, 60 and 90 min post *S. aureus* infection in presence of Riboflavin alone or in combination of CIP or AZM were also determined. A significant increase (*p* < 0.05) in NO production in the *S. aureus* infected macrophages was found at 30, 60 min and 90 min of phagocytic time when compared to control macrophages at level of signifcance. When the *S.aureus* infected macrophages were incubated in presence of only Riboflavin a significant reduction in the NO production at 30, 60 and 90 min was recorded (Fig. 5). The amount of NO production was further reduced significantly (*p* < 0.05) in presence of both Azithromycin or Ciprofloxacin when each were co-treated with Riboflavin at all three time points and compared with RIBO + SAM. Riboflavin treated groups also showed significant difference in the presence of only antibiotics treated plus infected groups. However, no significant difference was noticed when CIP with Riboflavin treated group were compared to AZM and Riboflavin treated group (Fig. 5).

### Effect of Riboflavin and antibiotic treatment on *S. aureus* infection-induced alteration in the activity of catalase enzyme by murine peritoneal macrophages

**Fig. 6:**
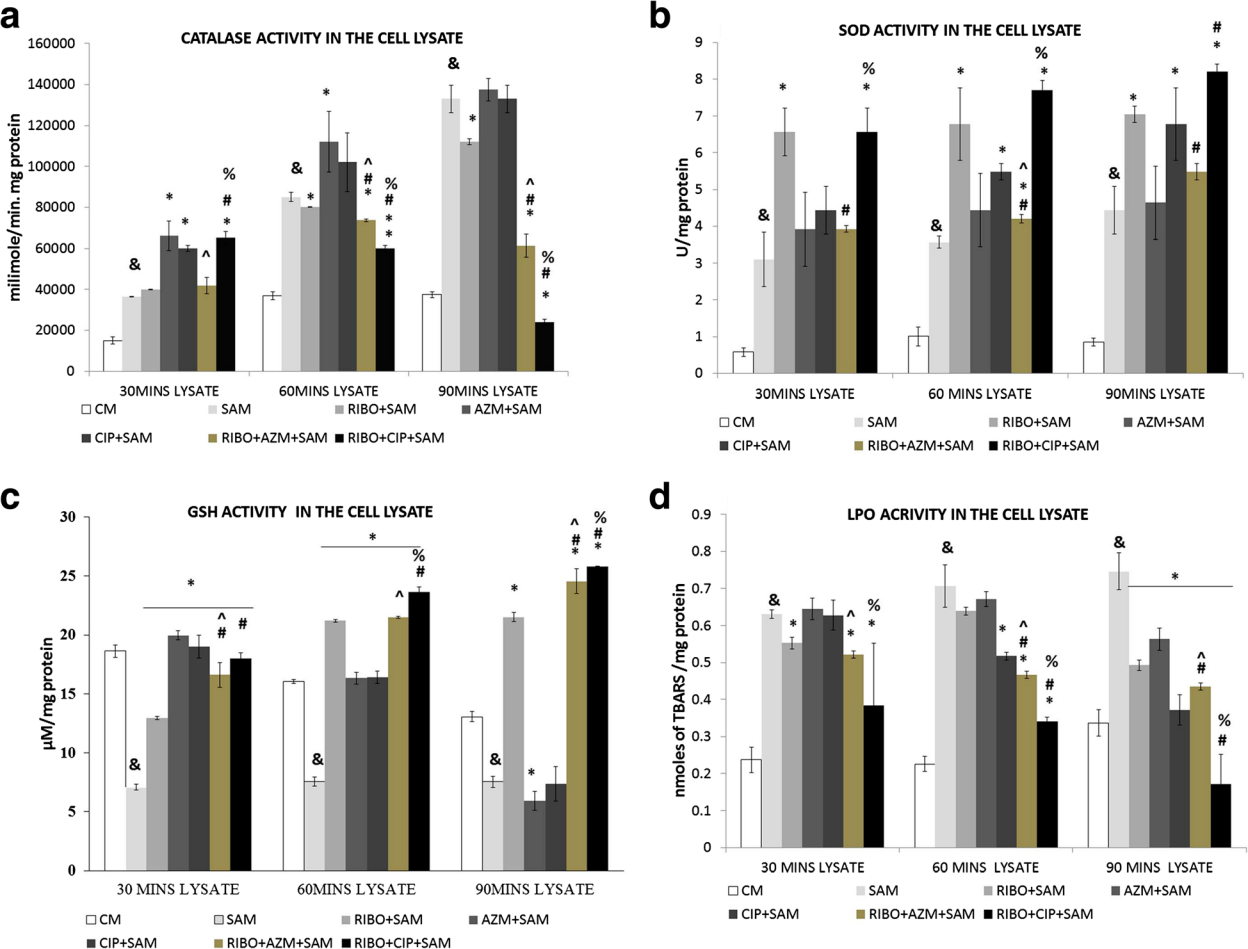
Effect of Riboflavin and antibiotic treatment (AZM and CIP) on *S. aureus* infection induced alteration on the activity of catalase, SOD enzyme and GSH, LPO level in the lysate of murine peritoneal macrophages. The lysate was recovered after time-dependent phagocytosis in the presence or absence of RIBO or antibiotic treatment, as described in the “Methods and materials” section, and were used to determine the catalase (**a**), SOD enzyme activity (**b**), GSH level (**c**), and LPO content (**d**) in the supernatant. Catalase enzyme activity was measured in the presence of 15 μmoles of H_2_O_2_/mLof phosphate buffer and is expressed in terms of mmol/min mg protein (**a**); SOD enzyme activity was expressed in terms of SOD units/mg protein (**b**); GSH activity was expressed in terms of μM/mg protein (**c**); and LPO content was expressed in terms of nmol of thiobarbituric acid-reactive substances (TBARS) (**d**) Results were shown as mean ± SD of three independent experiments. CM, Control macrophage; SAM, *S. aureus* infected macrophages; RIBO + SAM, Riboflavin pretreated + *S. aureus* infected macrophages; AZM + SAM, Azithromycin treated *S.aureus* infected macrophage; CIP + SAM, Ciprofloxacin treated *S.aureus* infected macrophage; RIBO + AZM + SAM, Riboflavin and Azithromycin pretreated macrophages exposed to *S. aureus*. RIBO + CIP + SAM, Riboflavin and Ciprofloxacin pretreated macrophages exposed to *S. aureus*; &, significant difference with respect to CM *, significant difference with respect to SAM; #, significant difference with respect to Riboflavin (RIBO) plus *S. aureus* infected macrophages at *P* < 0.05 level of significance. ^, significant difference with respect tociprofloxacin (CIP) or % Significant difference with respect to Azithromycin (AZM) plus *S. aureus* infected macrophages

Bacterial catalase played an important role in the intracellular survival of *S. aureus* in murine peritoneal macrophages, we became interested to find out whether there occur any changes in these antioxidant enzyme activities after treatment of *S. aureus* infected macrophages with riboflavin in presence or absence of antibiotics AZM and CIP. A marked increase (*p* < 0.05) in the activity of catalase enzyme was observed in the cell lysate of control macrophages at 30, 60 and 90 mins when were compared to *S. aureus* infected macrophages. Significant reduction in the activity of catalase enzyme was found when infected macrophages were treated with Riboflavin, though only at 90 min. A significant decrease in catalase enzyme activity (*p*< 0.05) was observed in the cell lysate of *S. aureus* infected macrophages when treated with AZM or CIP along with riboflavin than that of only Riboflavin treated but *S. aureus* infected macrophages (Fig. 6a).

### Effect of Riboflavin and antibiotic treatment on *S. aureus* infection-induced alteration in the activity of superoxide dismutase (SOD) enzyme by murine peritoneal macrophages

On the basis of above finding, we further assessed the SOD enzyme activity that was increased in case of *S. aureus* infected macrophages when compared with the control group. However, when the *S.aureus* infected macrophages were incubated in presence of only Riboflavin a significant (*p* < 0.05) increment in the activity of SOD enzyme at 30, 60 and 90 min was recorded in respect to only infected group (Fig. 6b). A significant increase in the activity of SOD enzyme was noticed at 60 mins when Azithromycin or Ciprofloxacin were both co-treated with Riboflavin and increase in 90mins was noticed only at CIP and RIBO treated group when all these were compared to Riboflavin treated *S.aureus* infected macrophages at (*p* < 0.05) level of significance (Fig. 6b).

### Effect of Riboflavin and antibiotic treatment on *S. aureus* infection-induced alteration in the GSH content by murine peritoneal macrophages

The amount of GSH content in the lysate was estimated. As shown in the Fig. 6c, a significant decrease (*p* < 0.05) in the GSH content of infected macrophages was noticed when compared to control macrophages. The increase in the GSH content in the lysate of Riboflavin treated macrophages at 30, 60 and 90 min of post infection with *S. aureus* were found to be significant (*p* < 0.05) when compared to the GSH content after time-dependent phagocytosis by the macrophages received no Riboflavin. However, addition of Riboflavin along with antibiotics to the peritoneal macrophages followed by infection of with *S. aureus* led to significant increase (*p* < 0.05) in the GSH content in lysate at 60 min and 90 min of incubation with CIP and at 90 mins with AZM treated group when compared to that of GSH level in Riboflavin treated plus *S. aureus* infected macrophages (Fig. 6c).

### Effect of Riboflavin and antibiotic treatment on *S. aureus* infection induced alteration in the LPO level in the lysate by murine peritoneal macrophages

Amount of LPO content in the lysate of *S. aureus* infected macrophages were significantly higher (*P* < 0.05) than that of control sets at 30, and 60 and 90 mins after *S. aureus* infection. A significant decrease of LPO level in the lysate of Riboflavin pre-treated macrophages were observed (*P* < 0.05) at 30 and 90 mins post-incubation when compared with the only *S.aureus* infected macrophages (Fig. 6d).

Macrophages pretreated either with CIP or AZM alone before *S. aureus* infection also showed decreased LPO content in the lysate in comparison to the amount of LPO level by the only *S. aureus* infected macrophages at 90 mins. Administration of either CIP or AZM to the Riboflavin pre-treated macrophages before *S. aureus* infection showed significant (*p* < 0.05) reduction in LPO content at 60 and 90 mins when compared with Riboflavin plus *S. aureus* infected macrophages (Fig. 6d). The LPO content was also significantly (*p* < 0.05) lowered at all time points when Riboflavin pretreated macrophages were incubated with CIP than AZM before *S. aureus* infection.

### Effect of Riboflavin and antibiotics AZM and CIP on the glutathione reductase activity of *S. aureus* infected murine peritoneal macrophages

The activity of glutathione reductase enzyme in the lysate was estimated. As shown in Table 3, the increase in the activity of glutathione reductase enzyme in the lysate at 30, 60 and 90 min post infection with *S. aureus* from Riboflavin pre-treated macrophages were found significant (*p* < 0.05) when compared to the activity of glutathione reductase enzyme after time-dependent phagocytosis by macrophages treated without Riboflavin. However, addition of Riboflavin along with antibiotics AZM or CIP to the peritoneal macrophages followed by infection with *S. aureus* led to significant increase (*p* < 0.05) in the activity of glutathione reductase enzyme in the lysate at 60 and 90 min of CIP treated macrophages and only in 90 mins in the AZM treated group when compared to the activity of glutathione reductase enzyme of Riboflavin treated *S. aureus* infected macrophages (Table 3).

**Table 3:** Glutathione reductase enzyme activity in the cell lysate

Groups	Glutathione reductase enzyme activity (nmol NADPH/min/mg of protein)		
	30 min	60 min	90 min
CM	0.066 ± 0.001	0.009 ± 0.0001	0.034 ± 0.01
SAM	0.113 ± 0.04^a^	0.263 ± 0.029^a^	0.355 ± 0.05^a^
RIBO + SAM	0.432 ± 0.05^b^	0.435 ± 0.04^b^	0.749 ± 0.090^b^
AZM + SAM	0.058 ± 0.005	0.066 ± 0.001^b^	0.118 ± 0.021^b^
CIP + SAM	0.035 ± 0.001^b^	0.423 ± 0.007^b^	0.073 ± 0.008^b^
RIBO+ AZM + SAM	0.457 ± 0.09^b, c^	0.491 ± 0.034^b, c^	1.023 ± 0.022^b, d, c^
RIBO+ CIP + SAM	0.265 ± 0.04^b, e^	0.529 ± 0.021^b^, ^d, e^	1.561 ± .127^b, d, e^

The activity of Glutathione reductase enzyme was determined following the oxidation of NADPH to NADP+ during the reduction of oxidized Glutathione and the results were expressed as nmole NADPH/min/mg of protein. Results were shown as mean ± SD of three independent experiments

*CM*, Control macrophage, *SAM S. aureus* infected macrophages, *RIBO + SAM* Riboflavin pretreated + *S. aureus* infected macrophages, *AZM + SAM* Azithromycin treated *S.aureus* infected macrophage, *CIP + SAM* Ciprofloxacin treated *S.aureus* infected macrophage, *RIBO + AZM + SAM* Riboflavin and Azithromycin pretreated macrophages infected with *S. aureus*, *RIBO + CIP + SAM* Riboflavin and Ciprofloxacin pretreated macrophages infected with *S. aureus*

^a^significant difference with respect to CM

^b^significant difference with respect to SAM

^c^significant difference with respect to ciprofloxacin (CIP)

^d^significant difference with respect to Riboflavin(RIBO) plus *S. aureus* infected macrophages at *P* < 0.05 level of significance

^e^Significant difference with respect to Azithromycin (AZM) plus *S. aureus*infected macrophages

### Effect of Riboflavin and antibiotic treatment on *S. aureus* infection-induced TNF- α, IFN-γ, IL-6, IL-1β and IL-10 and chemokine MCP-1 production by murine peritoneal macrophages

**Fig. 7:**
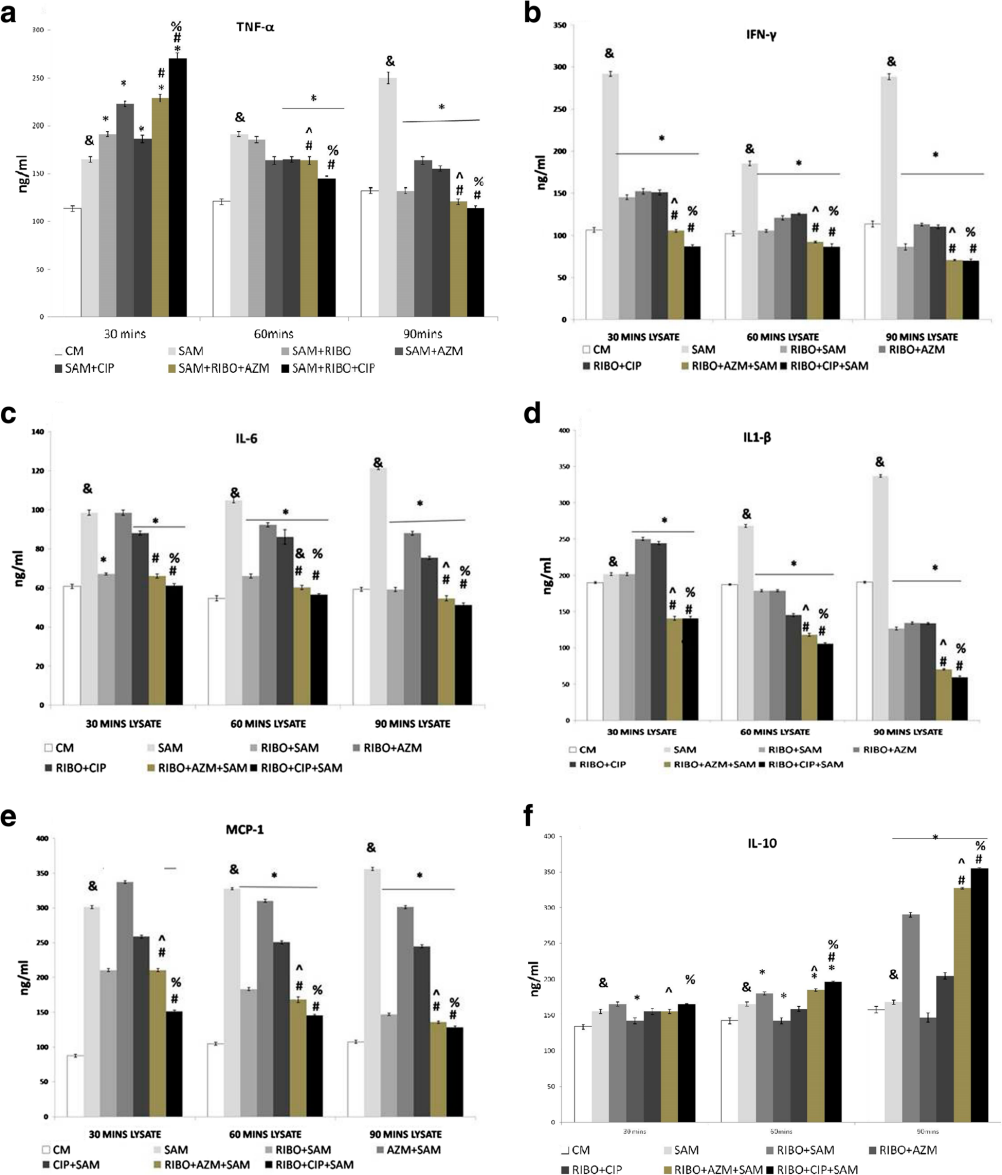
Levels of cytokines released in the supernatant as determined by ELISA. Levels of tumour necrosis factor-alpha (TNF-α) (**a**), interferon-gamma (IFN-γ) (**b**), IL-6(**c**), IL-1β (**d**), MCP1(**e**) and IL-10 (**f**) in the supernatants collected after 30, 60 and 90 min of *Staphylococcus aureus*–infected macrophages in the presence or absence of Riboflavin or antibiotics were determined by utilizing ELISA according to the manufacturer’s recommendations and were expressed from triplicate experiments. &, significant difference with respect to CM *, significant difference with respect to SAM; #, significant difference with respect to Riboflavin(RIBO) plus *S. aureus* infected macrophages at *P* < 0.05 level of significance.^, significant difference with respect to ciprofloxacin (CIP) or % Significant difference with respect to Azithromycin (AZM) plus *S. aureus* infected macrophages

Cytokine levels showed a significantly (*P* < 0.05) decreased level of TNF-α (Fig. 7a), IFN-γ (Fig. 7b), IL-6 (Fig. 7c), IL-1β (Fig. 7d) in the media (culture supernatant) with the increase of phagocytic time in the presence of Riboflavin when compared to the cytokine levels after time–dependent phagocytosis with Riboflavin non-treated but *S. aureus* infected macrophages. Macrophages that were pretreated either with CIP or AZM alone before *S. aureus* infection also showed reduced level of TNF-α, IFN-γ, IL-6, IL-1β in the supernatant at 60 and 90 mins in comparison to the amount of these cytokines released by the only *S. aureus* infected macrophages. These cytokine production was further decreased significantly (*p* < 0.05) when in addition to Riboflavin, macrophages were further incubated with AZM or CIP at 60 and 90 mins compared to only Riboflavin treated but *S. aureus* infected peritoneal macrophages. However, the level of IL- 10 increased significantly in the medium at 60 and 90 min when Riboflavin pretreated macrophages were incubated with CIP before *S. aureus* infection in comparison to Riboflavin plus *S. aureus* infected macrophages (Fig. 7f).

Chemokine MCP1 also showed a significantly (*p* < 0.05) marked reduction in its level in presence of Riboflavin when compared to Riboflavin non treated macrophages at 30, 60 and 90 mins respectively (Fig. 7e). The production of MCP1 was further reduced significantly (*p* < 0.05) in presence of antibiotics AZM or CIP when compared to only Riboflavin treated but *S.aureus* infected peritoneal macrophages (Fig. 7e).

### Effect of Riboflavin alone or with antibiotics AZM and CIP on the expression of COX-2 in peritoneal macrophages of Swiss albino mice

**Fig. 8:**
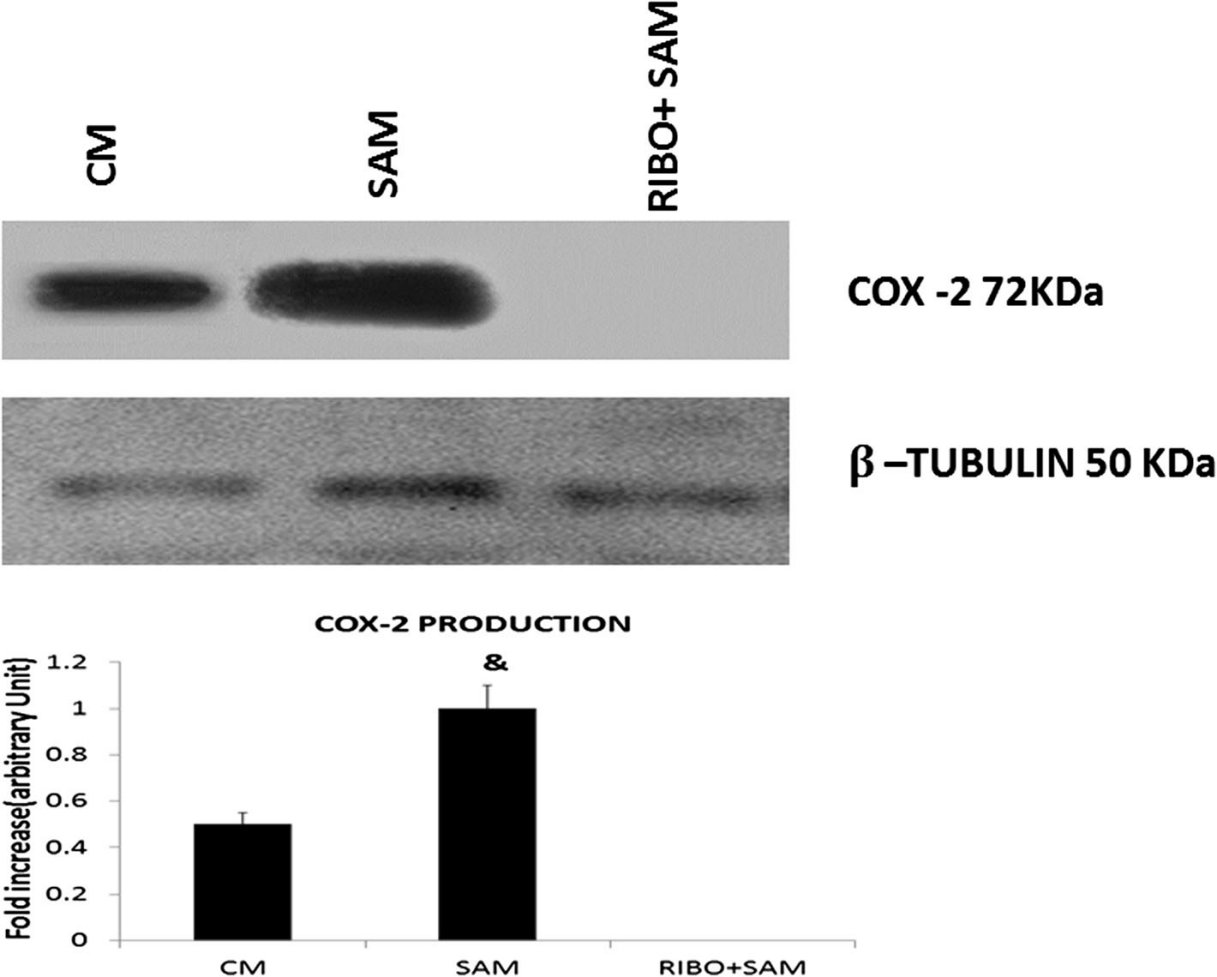
Effect of Riboflavin and antibiotics (AZM and CIP) on the expression COX2 analyzed by Western blot. The results in this Fig. 8 showed the expression of COX 2 protein in peritoneal macrophages after infection with *S. aureus* from a set of triplicate experiments. All the samples were probed with β-tubulin to show equal protein loading. ‘&’ represents significant difference with respect to CM (Control macrophage) at *p* < 0.05 level of significance

The expression of the Cox 2 was studied in murine peritoneal macrophages exposed to *S. aureus* when treated with either Riboflavin alone or in combination of antibiotics AZM or CIP recovered after 90 min of phagocytosis to get a wider overview of the Cox2 enzyme which play a pivotal role in inflammation. Data presented in Fig. 8 showed increased expressions of Cox2 in the peritoneal macrophages exposed to *S. aureus* when compared to the control macrophages at *p* < 0.05 level of significance*.* No, detectable band of COX2 was observed when peritoneal macrophages were treated with Riboflavin either alone or with AZM or CIP (Fig. 8).

## Discussion

The treatment of *S. aureus* infections has been increasingly problematic due to the high prevalence of multi-antibiotic-resistant strains, such as methicillin-resistant *S. aureus*, [29] and the emergence of vancomycin-resistant *S. aureus s*trains. As an alternative to traditional antibiotics in an era of increasing bacterial resistance, new attention has focused upon development of agents that can effectively disarm the pathogen burden and allow clearance by the host. The aim of this study was to determine the intracellular anti-staphylococcal activities of antibiotics CIP and AZM along with the presence of Riboflavin an established modulator of macrophage function to boost pathogen clearance and enhance longevity of the host cell by reducing cell death. It has been reported that Riboflavin at a concentration of more than 25 μg/ml could enhance macrophage function in-vitro [30]. Our result of recovered or uptaken Riboflavin concentration after treatment of macrophage with 100 μg/ml depicts that intake of Riboflavin by macrophages is more than 40 μg/ml (Table 1) which according to previous reports is more than the threshold value required for macrophage activation in-vitro. Riboflavin uptake is enhanced when macrophages were exposed to *S. aureus* and this uptake is incubation time dependent, i.e., 90mins incubation resulted in more Riboflavin intake than 60mins of incubation time. From our experiment of Riboflavin concentration inside the cell we found that the use of antibiotics AZM and CIP further enhanced Riboflavin concentration (Table 1). It has been reported that formation of intra mitochondrial FAD requires transport of Riboflavin from the cytosol into mitochondria via mitochondrial flavokinase. Because FAD itself does not traverse membranes, FAD requires degradation back to Riboflavin [31]. Therefore trafficking of Riboflavin inside the macrophages could be expected.

From the bacterial CFU count of *S. aureus* recovered after phagocytosis by Riboflavin pre-treated murine peritoneal macrophages, where, significantly lower number of bacteria has been found in comparison to Riboflavin untreated *S. aureus* infected macrophages, indicating the immunomodulatory role of Riboflavin and the presence of antimicrobial property in azithromycin and ciprofloxacin further aided in the clearance of the pathogen. Confocal imaging also depicted more engulfment in presence of Riboflavin alone or with AZM or CIP. Previous results indicated that the phagocytic ability of the macrophages may be significantly limited in macrophages with Riboflavin deficiency [8]. Therefore, a lower amount of viable intracellular bacteria directly correlated with increased engulfment and subsequent intracellular clearance in the given experimental set up. Taken together Riboflavin pretreated cells were active enough to kill the bacteria in presence of antibiotics. Since treatment with antibiotics and Riboflavin did not exert any toxic effect on macrophage and thus has no effect on the viability of macrophages therefore, reduced amount of viable intracellular bacteria could not be expected from decreased amount of extracellular bacteria or from an impaired phagocytic activity of macrophages due to the treatment with antibiotics and Riboflavin. However, we have not tested whether the treatment of antibiotics used and Riboflavin has any effect directly on the growth of *S. aureus*.

Undoubtedly with the elicitation of macrophages function in presence of Riboflavin (Table 1), reactive oxygen species like H_2_O_2_ and superoxide anion elevation has been found which give the ability to macrophages to destroy ingested cells. Riboflavin deficient macrophages may exhibit an impaired ability of phagocytosis and killing of ingested particles [32]. Decreased levels in ROS were observed when infected macrophages are treated with antibiotic alone. It has been shown that macrolides like AZM are able to inhibit the production of ROS from neutrophils and this is incubation time dependent [33], which has been suggested to be partly because of the stabilization of cell membrane. Macrolides attenuate the membrane-destabilizing effect of bioactive phospholipids, such as lysophosphatidylcholine, platelet-activating factor (PAF), and lyso-PAF, and this is accompanied by a dose-related inhibition of superoxide production. Interference with phospholipase/phosphatidic acid phosphohydrolase may also decrease superoxide generation by phagocytes [34]. The kinetics study of O_2_^−^ and H_2_O_2_, production in the lysate of macrophage cultures has revealed higher concentration of these anions in CIP and Riboflavin treated infected macrophages but lesser in the RIBO and AZM-treated infected macrophages after incubation. The phagocytosis of microorganisms activates oxidase dependent on adenine dinucleotide phosphatase (NADPH), which induces the production of high levels of superoxide, H_2_O_2_ a process commonly denominated as respiratory burst.

The enzyme Riboflavin Kinase converts Riboflavin to FMN and FAD. The crucial enzyme for ROS production i.e. NADPH oxidase enzyme complex depends on FAD which is one of the NADPH oxidase component [14, 35], therefore, elevated H_2_O_2_ and O_2_^−^ production in Riboflavin pretreated macrophages obtained in this study is also relevant and supported by earlier studies Fig. 3a, b]. Superoxide anion does not diffuse across membrane efficiently and is rapidly dismuted to H_2_O_2_ by SOD. However, H_2_O_2_ can diffuse more freely and causes direct oxidative damage to many pathogens. In the early innate immune responses, H_2_O_2_ kills bacteria through classic ROS respiratory burst.

Further elaboration of ROS, the effecter molecules is documented in the addition of Ciprofloxacin along with Riboflavin as it stimulates the oxidative burst in monocyte and macrophages. Azithromycin when combined with Riboflavin elicits the function of macrophage but lower in comparison to Riboflavin when combined to Fluroquinolone signaling [36]. In our study pre-treatment of macrophages with antibiotics does not lead to residual toxic effects on macrophages, and the effects are due to changes in response to live cells because in our results by FACS analysis of ROS a significant increase in ROS production on addition of antibiotic CIP was documented which cannot be attributed by dead cells.

Our data suggest that Riboflavin treatment diminishes the nitric oxide production by mouse peritoneal macrophages when exposed to *Staphylococcus aureus*. Suppression of nitric oxide induction and pro-inflammatory cytokines by novel proteasome inhibitors has been studied in various experimental models as in LPS stimulated RAW 264.7 macrophage [10]. Role of Riboflavin as a naturally occurring proteasome inhibitor has been extracted from literature according to which Riboflavin is potent inhibitors of chymotrypsin-like activity of 20S subunit of rabbit muscle proteasomes and mouse immune proteasome and its activity depends on protease site LMP7 and other signaling pathways [10].

In our study of thioglycolate elicited murine peritoneal macrophage which were infected by *S. aureus* the pathway by which Riboflavin is suppressing the NO production is through increasing cellular levels of PI-κB and decrease nuclear translocation of NF-κB, further decreasing degradation of ubiquinated PI-κB by the proteasome, and this inhibition by Riboflavin results in decreased translocation of NF-κB to the nucleus [10]. It has been reported that low levels of H_2_O_2_ can enhance NF-kB activity and higher H_2_O_2_ inhibited its activity [37]. The overall effect of NO and ROS on NF-kB activity follows a biphasic pattern and many of these observations are cell and mechanism dependent [38]. In addition, lower NO produced by *S. aureus* stimulated macrophages (Fig. 5) can combine lesser with superoxide to diminish the generation of additional product with enhanced toxicity, such as peroxynitrite, suggesting protection from more harmful ROS in presence of Riboflavin at 60 and 90 mins post incubation [39, 40].

It has been suggested that bacteria that has survived avoiding the host immune response must have to scavenge the host derived O_2_^−^ and H_2_O_2_ by enhancing bacterial SOD and catalase enzyme activity. Higher amount of SOD enzyme activity might have counteracted with the increased superoxide radical and H_2_O_2_, as evidenced by the increased SOD activity in lysate of macrophages exposed to *S. aureus* from Riboflavin treated macrophages. The increased SOD activity (Fig. 6b) in lysate, therefore, neutralizes the bactericidal activity of host macrophage derived O_2_^−^ by converting it into H_2_O_2_, another oxygen metabolite having potential antimicrobial activities whereas, decreased catalase activity (Fig. 6a) in the lysate of macrophages exposed to recovered *S. aureus* from Riboflavin treated macrophages suggests enhanced phagocytic capacity of the macrophages. Lesser Catalase and more SOD enzyme activity has also been documented when in addition to Riboflavin, cells were further co-treated with Ciprofloxacin at 60 and 90 mins of incubation. Macrolide antibiotics like AZM are also known to alter the physiological redox homeostasis leading to oxidative stress and lipid peroxidation [41]. This induction of oxidative stress may further reduce the catalase activity in 60 and 90 mins post incubation in presence of Riboflavin (Fig. 3a). Reports also suggests that in acute infection i.e., for 60 and 90 mins Ciprofloxacin increases the SOD activity leading to more production of H_2_O_2_ from more generated superoxide anion [42]. A study investigated the effect of Riboflavin therapy on diabetic cardiomyopathy documented that Riboflavin can increase SOD activity in the heart tissue [43]. So, increase in SOD activity in all Riboflavin treated groups can be attributed to antioxidant property of Riboflavin. Earlier it was demonstrated that TNF-α mediated activation of NF-κB Pathway may be inhibited by transient over expression of cytosolic SOD1 or mitochondrial SOD 2 but not catalase [44].

Glutathione is the master component of the antioxidant defenses in the cell. Both cellular activities of glutathione reductase and concentration of reduced glutathione are markers of flavin status and FMN and FAD participate in a range of redox reactions including the Glutathione system Our results demonstrate an increase in both Glutathione and Glutathione reductase activity when macrophages were stimulated in presence of Riboflavin at 60 and 90 mins of incubation. Presence of Riboflavin at a dose of 100 μg/ml converted it to FAD which may acted as a co-enzyme to increase glutathione reductase activity at 60 and 90 mins of incubation in the cell lysate [Table 3]. Further activation of glutathione reductase might have increased the reduced glutathione content in the cell as evident from our study at 60 and 90 mins of incubation (Fig. 6c) [45]. FAD transports hydrogen from NADPH to oxidised glutathione to convert it into the reduced form [46]. Reduced glutathione acts as an endogenous antioxidant in different cell types function of which can be enhanced by the activity of Riboflavin.

Increase of GSH and decrease of LPO in Riboflavin treated groups can be indicators of reduction of oxidative stress, as lipid peroxidation is a potent agent for cellular damage which acts by inhibiting membrane enzymes and receptors, depreciation of LPO and augmentation of GSH is more prominent when Riboflavin is combined with AZM/CIP (Fig. 6c and d).

The defense of an organism depends on the recognition of harmful stimuli followed by appropriate activation of innate and adaptive immune responses, leading to cytokine production. Several cytokines have pro-inflammatory actions that drive the innate immune response, cause inflammation and activate adaptive immune responses. During infection and sepsis elevated levels of pro-inflammatory cytokines, e.g. TNF-α and IFN- γ have been reported [47]. Pro-inflammatory cytokines like TNF-α, IL-1β, and IL-6 are inducer of systemic inflammation [20] and can be detrimental to host cell inducing hemodynamic shock and cell death. Our research has focused primarily on macrophages because they are highly sensitive to Riboflavin stimulation and respond by reducing production of TNF-α, IL-1β, IL-6 and IFN-γ.

TNF-α is a multifunctional pro-inflammatory cytokine that belongs to the tumor necrosis factor (TNF) super family [48]. Increased TNF-α from the Riboflavin plus CIP or Riboflavin plus AZM treated macrophages at early infection (at 30 min) indicating bacterial killing and better efficacy of both this antibiotics in presence Riboflavin (Fig. 7a). However, suppression of TNF-α level at late stage of infection by both the antibiotics in presence of Riboflavin indicates the combination to be effective in down regulating inflammation with increasing time which was beneficial to the host cell. Suppression of IL-1β, IFN-γ as well as IL-6 (Fig. 7d, b and c) by the Riboflavin and CIP or AZM treated macrophages at late stage of infection further confirms their anti-inflammatory nature. Macrolide antibiotics reportedly repress the production of pro-inflammatory cytokines in macrophages [49]. Apart from the microbicidal activity of fluroquinolone Ciprofloxacin, reports also pictures CIP also a potent immunomodulatory agent [50]. As already discussed Riboflavin is a potent protease inhibitor and this inhibitory effect governs the TNF-α signaling pathway through NF-κβ. Classical NF-κβ consist of heterodimers of p65 and p50 and these are the potent activators of plethora of general proinflammatory cytokines such as IL-6, TNF-α etc. In an inactive cell p50/65, is maintained in the inactive state in the cytoplasm of cells when it is bound to I-κB. Riboflavin has the capacity to inhibit proteasome activity in conjunction with their capacity to increase cellular levels of PI-κB and decrease nuclear translocation of NF-κB, suggests that the mechanism by which these agents suppress production of TNF-α and NO, involves decreased degradation of ubiquinated P-IκB by the proteasome, resulting in depressed translocation of NF-κB to the nucleus. Thus, Riboflavin exerts is anti-inflammatory effects by inhibiting NF-κB activation [10]. Based on these reports, the capacity to increase cellular levels of P-IκB and subsequently decrease nuclear translocation of NF-κB, we hypothesized that Riboflavin possibly down-regulate the NF-κB pathway. Given the important role of TNF-α in sepsis such suppression by combination therapy could be a therapeutic effect [51]. It is also known that exposure of staphylococci to beta-lactam antibiotics greatly enhances the release of bacterial cell wall fragments that have strong proinflammatory properties, such as peptidoglycan and lipoteichoic acid [52, 53], resulting in the induction of higher TNF-α levels from monocytes [54]. Previous reports demonstrated the clinical benefits of antibiotic and TNF-α inhibitor combination therapies relating to both arthritis severity and bone destruction in addition to reductions in the mortality rate by a combination therapy in experimental *S. aureus*–induced septic arthritis [55]. Recently we have observed that certain interaction exists between TNF-α and MMP-2 during the course of septic arthritis in *S. aureus* infected mice, hence further experiments are warranted to figure out the impact of TNF-α on MMP-2 activation via receptors for TNF-α (TNFR-I and TNFR-II) during *S. aureus* induced septic arthritis [56].

Among the pro-inflammatory cytokine milieu, anti-inflammatory cytokine IL-10 was also evaluated and it was found that early infection in presence of Riboflavin did not effect this anti-inflammatory cytokine, but at 60 and 90 mins of post incubation IL-10 was found to be increasing in presence of Riboflavin [57] and this effect was more prominent in acquaintances of Riboflavin with AZM and CIP (Fig. 7f).

Riboflavin is also known to modulate MCP-1 function which is a potent chemoattractant and a regulatory mediator involved in a variety of inflammatory diseases [58]. Evidences further support the action of fluroquinolones and AZM on suppression of MCP1 [59]. Our report shows a reduction in the value of this chemo-attractant in Riboflavin treated groups. Further in comparison to only Riboflavin treated group, combination with AZM or CIP further down regulated production of chemokine MCP 1 (Fig. 7e). Riboflavin action as an inflammation regulator has not only been justified but its role as an anti-inflammatory cytokine enhancer was also established from this study.

For a clear analysis of inflammation, a well known hallmark of acute inflammation COX2 expression was also analysed in our experimental setup [60]. A decrease in COX2 expression at 90 mins in all Riboflavin treated group was documented. NO has been found to modulate PGE2 synthesis in macrophage cell line and rat islet cells [61]. Our study shows declined production of NO which might be responsible for reducing COX2 expression as reduction in expression of iNOS tend to reduce expression of COX2 modulated by cytokines. IL-1β stimulated the expression of inducible nitric oxide synthase (iNOS) by β-cells [62]. So, from our study it can be speculated that lower level of IL-1β might have reduced the iNOS synthesis which by similar mechanism reduced COX2 expression. Reduction of inflammatory mediator like COX2 certainly illustrates Riboflavin as a potent inflammation regulator. In the in-vivo system treatment with azithromycin and Riboflavin completely eradicated the bacteria from blood and spleen. TNF-α, IFN-γ, IL-6, and MCP1 were down regulated by treatment with azithromycin and Riboflavin [63]. Treatment with Riboflavin also altered the antioxidant status which was further elaborated by using this combinational therapy in mouse peritoneal macrophage in the present study.

Caspase-1, however, seems to be uniquely involved in participating in the inflammatory response by cleaving the precursors of IL-1 beta, IL-18, and IL-33. Indeed, the rate-limiting step in inflammation due to IL-1 beta or IL-18 is the activation of caspase-1 [64]. Recently, a role for ROS has emerged in the activation of the NLRP3 inflammasome, one pathway for generation of active caspase-1 and secretion of mature IL-1β [65]. There is a distinct role for ROS in up-regulation of mRNA for inflammatory cytokines such as IL-1β and TNF-α. As, ROS upregulate Caspase 1 as well as IL-1β, So increase in ROS production on Riboflavin treatment might have upregulated Caspase 1 as well as IL-1β in early infections. In late infections reduction in IL-1β might have reduced Caspase 1 expression as there are reports that IL-1β secretion and reactive oxygen species (ROS) down-modulate IL-1β rather than activating it in phagocytes [66].

The isoalloxazine moiety of Riboflavin chelates metals such as cadmium, cobalt, copper, iron, molybdenum, manganese, nickel, silver, and zinc [67]. Some bound metals are easily oxidized and reduced and play a role in the formation of free radicals. Our study also evidenced an enhanced free radical production behind which iron chelation by Riboflavin may play a significant role. Iron has important role to play in the disease manifestation of a pathogen in the macrophage [68].

Pathogens release siderophores which chelate iron present in the macrophage for their own survival, but presence of Iron chelators like Riboflavin in the medium will chelate the iron and make it unavailable to the bacteria secreted siderophores, Thus depletion of Iron will hinder the pathogen’s replication and thus their survival will be at a stake.

It appears desirable to keep the concentration of pro-inflammatory products of pathogens in the tissue low during the whole course of an infection to decline host cell death. In clinical practice, a favourable outcome depends on the antibiotic treatment after hospital admission. Therefore, the reduction of potentially deleterious pathogen derived compounds by rapid initiation of an effective antibiotic therapy along with choosing compounds which synergistically act with antibiotics to boost the killing process and further do not release large amounts of pathogen products is a promising strategy to over -stimulate phagocytic cells and decrease tissue injury. It restricts host cell damage which depicts a protective approach of Riboflavin along with antibiotics like AZM and CIP.

Similar results were also found in case of bone marrow derived macrophages (BMDM) when pre-treated with Riboflavin and exposed to *S.aureus.* The phagocytic activity increased with the treatment of riboflavin along with increased ROS production leading to augmentation in the killing of pathogen. This comparative analysis of BMDM to peritoneal macrophages showed similar mechanism of action, confirms human relevance, or at least efficacy during initial infection conditions.

The use of these novel mechanisms can be implemented in targeted delivery of drugs through targeted drug carriers like microcapsules, liposomes and micelles etc. in in-vivo system. In arthritis, a diversity of organisms are present in deep infection, the dominant bacteria are Gram positive pathogens with *S. aureus*. Established treatments rely on the controlled release of antibiotics. Therefore, this targeted drug carrier will selectively and effectively localize pharmacologically active moiety of Riboflavin and CIP at pre selected target in therapeutic concentration while limiting its access to non-targeted cells thereby minimizing toxic effect and maximizing therapeutic index.

## Conclusion

**Fig. 9:**
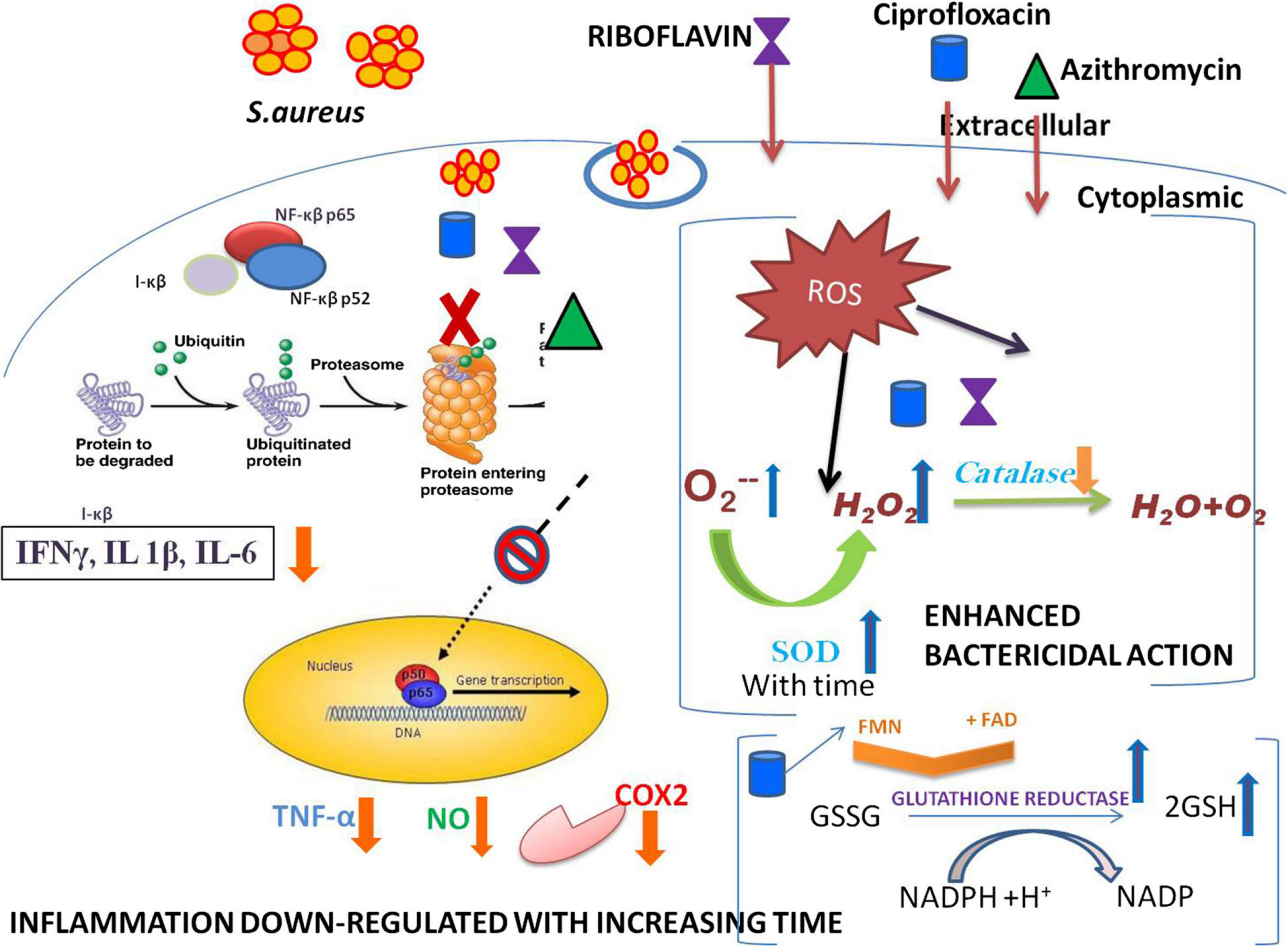
Schematic representation of the study. This study suggests a possible mechanism to augment the immunological response on use of Riboflavin with antibiotics, AZM and CIP, which is achieved by enhanced ROS, ensuring enhancement of pathogen clearance and by the antioxidant properties of Riboflavin protection of the host cell is ensured, thus helped in maintaining a balanced redox state in the cell. Riboflavin by its proteasome inhibitory action might down regulates the NF-κβ pathway, thus reducing pro-inflammatory cytokines, nitric oxide and COX2, which on one hand prevent infection and on other hand lowers inflammatory damage

In conclusion, our study suggests a possible mechanism to potentiate the immunological response on use of Riboflavin along with combinations of antibiotics, by enhanced ROS, ensuring augmentation of pathogen clearance and by the antioxidant properties of Riboflavin protection of the host cell is ensured, thus helped in maintaining a balanced redox state in the cell. Riboflavin by its proteasome inhibitory action might down regulates the NF-κβ pathway, thus reducing pro-inflammatory cytokines, nitric oxide and COX2, which ensures protection from infection on one hand and survival benefit of host cells from inflammatory damage on the other. The balance between the redox reactions and antioxidant system suggests a strong therapeutic intervention in serious bacterial infections associated with sepsis at least in our in-vitro system [Fig. 9].

## Abbreviations

AZM: Azithromycin
CIP: Ciprofloxacin
COX-2: Cyclogenase 2
CPCSEA: Committee for the purpose of control and supervision of experiments on animal
ELISA: Enzyme linked immunosorbent assay
iNOS: Inducible nitric oxide synthase
NF-kB: Nuclear factor -kappa beta
RIBO: Riboflavin
ROS: Reactive oxygen species
RPMI: Roswell Park Memorial Institute medium
SDS-PAGE: Sodium dodecyl sulphate polyacrylamide gel electrophoresis
